# Transcriptomic Analysis of circRNAs and mRNAs Reveals a Complex Regulatory Network That Participate in Follicular Development in Chickens

**DOI:** 10.3389/fgene.2020.00503

**Published:** 2020-05-15

**Authors:** Manman Shen, Tingting Li, Fuxiang Chen, Pengfeng Wu, Ying Wang, Lan Chen, Kaizhou Xie, Jinyu Wang, Genxi Zhang

**Affiliations:** ^1^College of Animal Science and Technology, Yangzhou University, Yangzhou, China; ^2^Jiangsu Key Laboratory of Sericultural Biology and Biotechnology, College of Biotechnology, Jiangsu University of Science and Technology, Zhenjiang, China; ^3^Jiangsu Institute of Poultry Science, Yangzhou, China

**Keywords:** follicle, transcriptome, circRNA, WGCNA, hub genes, *ESR1*

## Abstract

Follicular development plays a key role in poultry reproduction, affecting clutch traits and thus egg production. Follicular growth is determined by granulosa cells (GCs), theca cells (TCs), and oocyte at the transcription, translation, and secretion levels. With the development of bioinformatic and experimental techniques, non-coding RNAs have been shown to participate in many life events. In this study, we investigated the transcriptomes of GCs and TCs in three different physiological stages: small yellow follicle (SYF), smallest hierarchical follicle (F6), and largest hierarchical follicle (F1) stages. A differential expression (DE) analysis, weighted gene co-expression network analysis (WGCNA), and bioinformatic analyses were performed. A total of 18,016 novel circular RNAs (circRNAs) were detected in GCs and TCs, 8127 of which were abundantly expressed in both cell types. and more circRNAs were differentially expressed between GCs and TCs than mRNAs. Enrichment analysis showed that the DE transcripts were mainly involved in cell growth, proliferation, differentiation, and apoptosis. In the WGCNA analysis, we identified six specific modules that were related to the different cell types in different stages of development. A series of central hub genes, including *MAPK1*, *CITED4*, *SOD2*, *STC1*, *MOS*, *GDF9*, *MDH1*, *CAPN2*, and *novel_circ0004730*, were incorporated into a Cytoscape network. Notably, using both DE analysis and WGCNA, *ESR1* was identified as a key gene during follicular development. Our results provide valuable information on the circRNAs involved in follicle development and identify potential genes for further research to determine their roles in the regulation of different biological processes during follicle growth.

## Introduction

Follicle development is a complex biological process involving a series of events, such as follicle recruitment, follicle selection, and oviposition. The process of follicle development extends from the primordial follicles to the establishment of a well-organized follicular hierarchy and is an important factor in the maintenance of high reproductive efficiency in hens. An intact hen follicle consists of one oocyte and two main types of somatic cells, theca cells (TCs) and granulosa cells (GCs). Abundant blood vessels surround or penetrate the basement membrane and the granulosa layer (Yoshimura and Koga, 1982; Wulff et al., 2001). GCs have been extensively studied because they are easily obtained and cultured *in vitro* (Johnson, 2015). Research has shown that GCs in the granulosa layer are responsive to follicle-stimulating hormone (FSH) during follicle selection (Hocking, 2009), and are the primary sites of progesterone secretion. In preovulatory follicles, GCs are involved in yolk deposition (Kim et al., 2016). Several genes, such as *BMP15* (Elis et al., 2007), *GDF9* (Paradis et al., 2009), and *STAR* (Zhu et al., 2015), have been shown experimentally to play important roles in the proliferation and differentiation of follicles, and the regulation of the genes that encode them involves multiple signaling pathways, including the steroidogenic pathway (Lee et al., 1998), transforming growth factor β (TGF-β) signaling pathway (Zhu et al., 2014), and oocyte meiosis (Peng et al., 2018).

With the development of molecular biological techniques, TCs have been widely studied *in vivo* and *vitro* (Kang et al., 2017; Wang and Gong, 2017). Previous reports have shown that the main function of TCs in birds is the production of androgens and estrogens (Bahr, 1991; Lee et al., 1998), which differs from the situation in mammals. Molecules such as *CYP19A1* (Wang and Gong, 2017) and the hormone prostaglandin (Jia et al., 2010) promote the proliferation of TCs. The pathways involved in TC growth include the GPCR/cAMP signaling pathway and lipid/amino acid metabolism pathways in the horse (Donadeu et al., 2014) and the angiogenesis pathway in primates (Wulff et al., 2001).

Although GCs and TCs play different roles in follicle development, their involvement is interactive. Follicular atresia, the main process by which follicles are lost, is not only associated with GCs (Kim and Johnson, 2018) but also with TCs, albeit less strongly (Lebedev et al., 2006), and with the inadequate penetration of blood vessels among TCs (Kim et al., 2016).

An increased number of studies have demonstrated that transcriptional regulation is modulated by both protein coding RNAs and non-coding RNAs. Protein-coding RNAs play key roles in life processes, whereas non-coding RNAs can act in mediation roles during the expression of protein-coding RNAs. Non-coding RNAs have recently emerged as integral components of folliculogenesis (Cheng et al., 2017; Kang et al., 2017; Peng et al., 2018). Circular RNA (circRNA), single-stranded RNA which unlike the better known linear RNA, forms a covalently closed continuous loop, has become a research hotspot since Salzman et al. (2012) identified numerous circRNAs in 2012. Structure feature of circRNA display stability (Li et al., 2017) that are not easily degraded by RNase R (Alvaro Mercadal et al., 2015; Lu et al., 2015). They are also conserved across species (Jeck et al., 2013). The expression levels of circRNAs are tissue- and developmental-stage specific (Xu et al., 2017), and they are potential biomarkers of cancers, with diagnostic implications (Shang et al., 2019). With technical advances, thousands of circRNAs have been identified in the liver and embryonic muscle of chickens (Ouyang et al., 2017; Zhang et al., 2017; Chen et al., 2019), but there is only limited information on the circRNAs involved in follicle development, especially in chickens. However, the transcriptomic profiles of circRNAs in the follicles and ovary have been reported in mice (Jia et al., 2018), humans (Cheng et al., 2017; Cai et al., 2018), goats (Tao et al., 2017), and pigs (Guo et al., 2019). Research on mice has shown that estrogen signaling is upregulated in adult ovaries relative to that in neonatal ovaries (Jia et al., 2018). A study of ovarian senescence in humans showed that the differentially expressed circRNAs in aging ovaries compared with young ovaries were enriched in the steroid hormone biosynthesis and insulin secretion pathways, and that *circDDX10* may be a sponge for microRNA miR-1301-3p or miR-4660 in modulating the expression of the *SIRT3* gene, which may be associated with ovarian function (Cai et al., 2018). Another study of human GCs reported that circRNAs expressed during maternal aging may participate in glucose metabolism, ovarian steroidogenesis, etc. (Cheng et al., 2017). Quan and Li (2018) hypothesized that *ciRS-7* influences oocyte maturation by regulating epidermal growth factor receptor (*EGFR*) expression. A study in goats showed that the target genes of circRNAs derived from the follicle may be involved in the GnRH signaling pathway, progesterone-mediated oocyte maturation, and the FoxO signaling pathway (Tao et al., 2017). The results of these studies suggest that circRNAs play functional roles in the ovary. However, there is only limited information on the roles of circRNAs during follicle development in chickens, which is a useful model in which to investigate reproductive diseases and follicular development (Bahr, 1991; Mellouk et al., 2018).

Although follicle development is a complex process involving multiples genes and interactions between GCs and TCs, no follicle-derived circRNAs that control its growth have been identified. In this study, we investigated the functions of circRNAs and mRNAs and their interactions in folliculogenesis.

## Materials and Methods

### Ethics Statement

All procedures involving birds were approved by the Animal Care Committee of Yangzhou University, Yangzhou, China (no. SYXK [Su] 2012-0029) and were conducted in accordance with the guidelines of the Chinese Ministry of Science and Technology. The birds were humanely killed as necessary and all animal suffering was minimized.

### Birds and Sample Collection

Jinghai Yellow chickens used in present study were sixteen parental generation population from Jiangsu Jinghai Industry Poultry Group Co, Ltd (Nantong, Jiangsu, China). The birds in this Company had been purified of Salmonella and Avian leukosis for more than 5 years. The positive rate was none in the present populations. Routine immunization procedures were used throughout the whole life of all the chickens. All hens were reared under natural light and were transferred to single cages from 16 weeks old, with 10% restriction of food and free access to water. Light was increased by 1 h per week until 16 h of light was provided. The laying nutrient levels content 11.3–11.92 MJ/kg metabolizable energy, 15.0–16.0% crude protein, 3.35% Ca and 0.45% P. Egg reproduction and body weight were recorded during the egg-laying period. The mean egg laying rate and the body weight of the birds used was 82.3% and 1983 ± 125 g, respectively. At 27 weeks of age, the birds sharing half-sib relationships were used for follicle collection. The hens with laying sequences of 5 were killed with 60–70% carbon dioxide, and the ovaries of each bird were immediately removed. The GCs and TCs were separated from three birds with eggs in their oviducts which were in three different physiological stages: (small yellow follicle [SYF], smallest hierarchical follicle [F6], and largest hierarchical follicle [F1] stages), according to the methods described by Gilbert and Eresheim (Gilbert et al., 1977; Eresheim et al., 2014). SYF which has a diameter about 4–8 mm is a stage of yolk accumulation beginning, with a yellowish appearance. The hierarchical follicles are numbered from the largest (F1, with a diameter about 40 mm) to the smallest (F6, with a diameter 9–12 mm), where F1 will be the next to ovulate (Johnson, 1990). F6 is a result of follicle selection from SYF, and F1 is a result of rapid proliferation from F6 after about 6 days. The tissues were placed immediately in liquid nitrogen and stored at −70°C.

### RNA Library Preparation

Total RNA was extracted with TRIzol Reagent (Invitrogen, Carlsbad, CA, United States), according to the manufacturer’s instructions. RNA contamination was checked on 1% agarose gels. The purity and integrity of the RNA were measured with a NanoPhotometer (Implen, United States) and an Agilent 6000 Nano Kit (Agilent Technologies, United States). The RNA concentrations were determined with a Qubit RNA Assay Kit in a Qubit 2.0 Fluorometer (Life Technologies, United States).

The total RNA from each tissue, with an RNA integrity number (RIN) > 8.5, was used as the input material for the RNA sample preparation. All total RNA samples were treated with the Epicentre Ribo-Zero^TM^ rRNA Removal Kit (Epicentre, Madison, WI, United States) to remove rRNA. Moreover, libraries of previously identified circRNAs were also digested with 3 U of RNase R (Epicentre). A total of 36 RNA libraries were constructed with the NEBNext^®^ Ultra^TM^ Directional RNA Library Prep Kit for Illumina^®^ (NEB, Ipswich, MA, United States), according to the manufacturer’s instructions. The index-coded samples were then clustered with the cBot cluster generation system using TruSeq PE Cluster Kit v3-cBot-HS (Illumina, United States). Finally, the libraries were sequenced on an Illumina HiSeq 4000 platform and 150-bp paired-end reads were produced. RNA-seq raw sequence reads from the follicle samples in current study can be accessed from PRJNA481176 and PRJNA511712 BioProject, available at the NCBI SRA repository (Supplementary Table S3E).

### Identification of Transcripts

We filtered the raw reads in the fastq format to remove those reads containing adapter, poly-N, or low-quality sequences. The Q30 scores and G + C contents of the clean data were calculated. For the genome-wide identification of circRNA transcripts, we aligned the clean paired reads to the reference genome Galgal 5.0 using Bowtie (Langmead and Salzberg, 2012). Two methods, find_circ (Memczak et al., 2013) and CIRI2 (Gao et al., 2017), were used to detect and identify circRNAs. Based on the features of circRNAs, we have summarized the characteristics of circRNAs in the chicken follicle. The features of circRNAs were determined based on their sequences.

To annotate the protein-coding transcripts, the clean reads were aligned to Galgal 5.0 with HISAT2 (Kim et al., 2019) using the parameter “—rna-strandness RF” and other default settings. The mapped reads were assembled with StringTie (v1.3.1) using a reference-based method (Pertea et al., 2016).

### Differential Expression Analysis

The raw counts of circRNAs were normalized as transcripts per million (TPM) with the following criteria (Zhou et al., 2010). To identify circRNAs differentially expressed in TCs and GCs, an analysis based on the negative binomial distribution was performed with the DESeq R package (1.10.1) (Love et al., 2014). The expression of protein-coding transcripts was calculated as the expected number of fragments per kilobase of transcript sequence per million base pairs sequenced (FPKM) with Cuffdiff (v2.1.1) (Trapnell et al., 2010). The differentially expressed circRNAs were detected with TPM. All transcripts with fold changes of >2 and *P*-adjusted < 0.05 were deemed to be differentially expressed. To study the relationships between the circRNAs and the corresponding host protein-coding genes, we analyzed this fold change in expression in GCs relative to TCs in different follicles.

### Co-expression of Transcripts

A weighted gene co-expression network analysis (WGCNA) (Langfelder and Horvath, 2008) was used to assess the potential functions of transcripts expressed in the granulosa layer or thecal layer during follicle development. Briefly, genes with the top 25% variance of expression values among samples were included (which included 7217 protein coding RNAs and 4504 circRNAs) as the input matrix for the WGCNA analysis. The best power was set to 12 to balance the scale-free property of the co-expression network (Supplementary Table S1). The cutreeDynamamic function was used with the parameters minModuleSize = 30, deepSplit = 2, pamRespectsDendro = F, and cutHeight = 0.3 to merge these modules, whose expression profiles were very similar. Gene significance (GS) was defined as the absolute value of the correlation between the gene and the differentiation stage. Module membership (MM) was defined as the correlation between the module eigengene and the gene expression profile. Hub genes were filtered with GS > 0.4 and MM > 0.8, and a bioinformatic analysis was performed in KOBAS (Xie et al., 2011). The top 200 connects based on the hub genes ranked by weighted values were visualized with Cytoscape (v3.6.1) (Shannon et al., 2003).

### Gene Ontology (GO) and Kyoto Encyclopedia of Genes and Genomes (KEGG) Enrichment

A GO enrichment analysis of the differentially expressed genes, the host genes of the circRNAs, and the hub genes was performed at the KOBAS. The GO terms or KEGG pathways with *P*-values < 0.05 or the top 20 terms are shown in the figures and tables.

### Validation of Transcripts

The splice sites of the circRNAs were first confirmed with divergent primers (Jeck et al., 2013) (Supplementary Table S2), which were designed with the “out-facing” strategy to exclude linear mRNAs by RNase R (Epicentre, NC, United States). The reverse transcription reaction was carried out with PrimeScript^TM^ RT Master Mix (Takara, Dalian, China), and the PCR products were tested with agarose gel electrophoresis. The PCR products were sequenced with Sanger sequencing at Sango Biotech Co. Ltd (Shanghai, China). Divergent primers and exon–exon-spanning primers were then used to confirm the expression levels of the circRNAs and their corresponding host genes on an ABI 7500 thermocycler (Life Technologies, United States). The expression levels of the transcripts were quantified with AceQ qPCR SYBR Green Master Mix (Vazyme, Nanjing, China), in a final volume of 20 μl, according to the user manual. Each reaction was performed in triplicate under the same experimental conditions: 95°C for 30 s and 40 cycles of 95°C for 5 s and 60°C for 34 s. The 2^–ΔΔ*Ct*^ method was used to calculate the gene expression levels, with β-actin as the reference gene.

## Results

### Summary of Transcriptome Profiles

The quality control parameters for the RNA-seq data are shown in Supplementary Table S3. A total of 864,689,266 and 828,121,626 raw reads of circRNA were obtained from the GCs and TCs, respectively. A total of 972,646,432 and 831,515,782 raw reads of linear RNA were obtained from the GCs and TCs, respectively. The percentages of reads that mapped to the chicken reference genome (Gallus-gallus-5.0/galGal5) were all >92%; the error rates were all <0.03%; and the Q30 scores were all >90%. Interestingly, the G + C contents of the circRNAs identified in the GCs or TCs were all >60%, whereas those of the linear RNAs were around 47%. The G + C contents in the TCs were higher than those in the GCs for both the circRNAs and linear RNAs.

**FIGURE 1:**
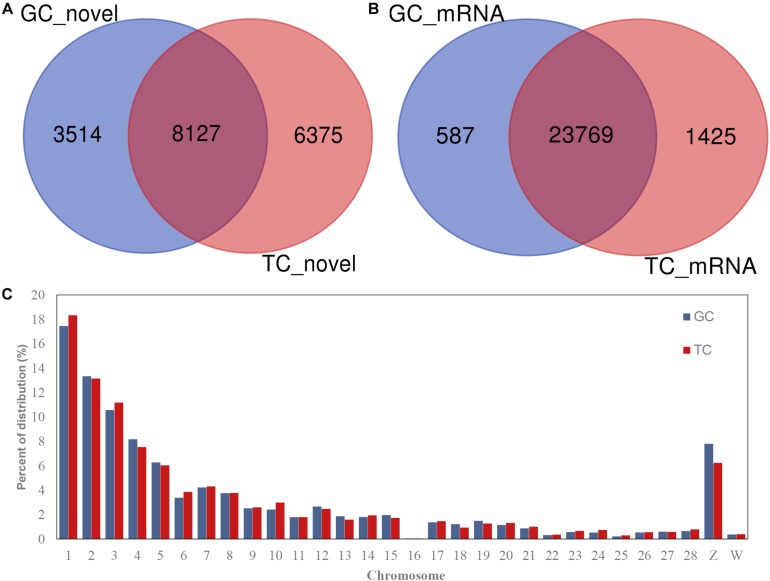
circRNAs and mRNAs detected in GCs or TCs. **(A)** Novel circRNAs detected in GCs and TCs; **(B)** mRNA identified in GCs and TCs; **(C)** circRNA distribution on chromosome. GCs, granulosa cells; TCs, theca cells.

A total of 18,016 novel circRNAs were detected in the GCs and TCs, 8127 (with red font in Supplementary Table S4) of which were abundant in both cell types (Figure 1A and Supplementary Table S4). A total of 25,781 mRNAs were identified in the GCs and TCs, 23,769 of which were detected in both cell types (Figure 1B). The distribution of circRNAs on chromosomes 1–28 and the Z and W chromosomes were analyzed, and showed that the longer the chromosome, the greater the percentage of circRNAs. Interestingly, the percentage distribution was lowest on chromosome 16 (Figure 1C).

Of the linear reads, ∼88.3% of the paired reads mapped to unique sequences in the chicken genome. Both read1 and read2 mapped to 44% of the reference genome. The numbers of reads that mapped to the “+” strand or “−” strand were nearly the same. The number of reads that mapped to splice sites was higher in the F1 follicles than in the F6 or SYF follicles (Supplementary Table S5a).

The features of the circRNAs were analyzed to clarify their roles in the follicle production process. The features of the circRNAs derived from GCs and TCs were almost the same in Supplementary Table S5. The source of the circRNAs, either coding sequences or intronic regions, was close to 40% (Supplementary Table S5b). The lengths of the circRNAs were predominantly 100–500 nt, with most in the range of 200–400 nt (Supplementary Table S5c). Almost a third of exonic-type circRNAs (38.44 and 40.39% in GCs and TCs, respectively) involved two exons, and about 27% of exonic-type circRNAs involved three exons. In brief, most exonic type circRNAs involved 1–5 exons (Supplementary Table S5d), with host gene lengths of >8000 kb (Supplementary Table S5e) and were separated by genomic distance of <10 kb (Supplementary Table S5f). The circularization of circRNA is affected by the length of the flanking intron, and our data show that the most frequent flank length (more than 40%) was 10^4^ kb (Supplementary Tables S5g–i).

### Differentially Expressed Transcripts

The numbers of differentially expressed (DE) transcripts are listed in Table 1, which shows that the number of DE circRNAs exceeded the number of DE mRNAs, and that DE mRNAs were only detected in the SYFG vs. SYFT comparison. A total of 625 DE circRNAs were identified in SYFG relative to SYFT, 271 of which were upregulated and 354 downregulated, whereas 145 DE mRNAs were detected in this comparison, with 37 regulated and 108 downregulated. In the F6 development stage, a total of 806 circRNAs were differentially expressed in GCs and TCs, but only five mRNAs were differentially expressed. Most DE circRNAs were detected in F1G vs. F1T comparison, 614 of which were upregulated and 828 downregulated.

**TABLE 1:** Numbers of differentially expressed transcripts in different comparisons of RNA-Seq experiments.

**Comparative group**	**circRNA**			**mRNA**		
	**DE^1^ circRNA**	**Up**	**Down**	**DE^1^ mRNA**	**Up**	**Down**
SYF^2^G^5^ vs. SYFT^6^	625	271	354	145	37	108
F6^3^G vs. F6T	806	416	390	5	4	1
F1^4^G vs. F1T	1442	614	828	1	0	1

*DE^1^, differentially expressed. SYF^2^, small yellow follicle; F6^3^, smallest hierarchical follicle; F1^4^, largest hierarchical follicle. G^5^, granulosa cell; T^6^, theca cell.*

**FIGURE 2:**
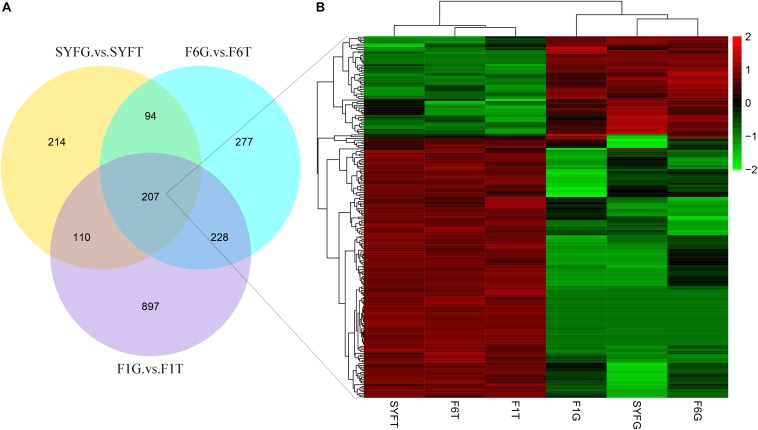
Differentially expressed circRNAs in venn diagram and heatmap. **(A)** Venn diagram of differentially expressed circRNAs; **(B)** heatmap of 207 pervasive circRNAs in three follicular development stage. SYF, small yellow follicle; F6, the smallest hierarchical follicle; F1, the largest hierarchical follicle. GCs, granulosa cells; TCs, theca cells.

The top DE circRNAs in the GCs and TCs are shown in Tables 2, 3, respectively. About 8/10 and 7/10 circRNAs were derived from protein-coding genes in the GCs and TCs, respectively. Host genes of DE circRNAs in the GCs included *SSPBP2*, *IGF1R*, *TOX2*, and *USP22*, whereas in the TCs, they included *IL1R1*, *MINDY4*, *FGFR2*, and *CRIIM1*. Notably, novel_circ_0002934 produced from the *RAB11A* gene was expressed at high levels in both GCs and TCs. A total of 2027 DE circRNAs were identified, 207 of which were abundantly expressed in the GCs and TCs at all three follicular stages (Figure 2A). However, their expression levels showed opposite trends in GCs and TCs (Figure 2B): in GCs, the expression levels were first clustered in two groups, SYFG and F6G, and then in F1G, whereas in TCs, the circRNAs were first clustered in F6T and F1T.

**TABLE 2:** Top 10 differentially expressed circRNAs in granulosa cells.

**circID**	**Splice length (nt)**	**Feature**	**Gene name**	**SYFG**	**F6G**	**F1G**	**SYFT**	**F6T**	**F1T**
novel_circ_0002240	288	Exon	*SSBP2*	4447.64	1002.17	111.02	1253.88	3100.16	1175.12
novel_circ_0002856	546	Exon	*IGF1R*	5213.18	3640.06	6267.09	1632.08	1703.10	1608.30
novel_circ_0002934	471	Exon	*RAB11A*	2701.79	3236.86	2108.44	7786.09	7939.65	8398.71
novel_circ_0004327	397	Intergenic	*–*	2820.19	2596.62	1903.66	423.28	334.98	281.45
novel_circ_0005320	394	Exon	*USP22*	11193.96	8445.37	2290.39	1489.48	1297.88	1155.95
novel_circ_0011474	406	Exon	*YBX3*	3125.14	1832.71	1332.86	476.27	392.72	464.48
novel_circ_0012077	312	Exon	*TOX2*	6239.09	3101.73	511.50	263.88	236.03	59.69
novel_circ_0013720	178	Exon	*EPT1L*	2792.32	1454.13	686.44	181.64	88.03	43.32
novel_circ_0013926	486	Intergenic	*–*	5767.53	2055.63	2158.72	735.60	655.13	447.61
novel_circ_0016978	318	Exon	*PTPN23*	4663.90	4328.23	2835.60	1686.27	2126.51	1269.76

*The latter six columns were referred to the Normalized TPM value. SYF, small yellow follicle; F6, smallest hierarchical follicle; F1, largest hierarchical follicle. G, granulosa cell; T, theca cell.*

**TABLE 3:** Top 10 differentially expressed circRNAs in theca cells.

**circID**	**Splice length (nt)**	**Feature**	**Gene name**	**SYFG**	**F6G**	**F1G**	**SYFT**	**F6T**	**F1T**
novel_circ_0000076	278	Intergenic	*–*	370.84	164.00	458.69	4299.98	6606.55	2860.13
novel_circ_0002589	715	Exon	*ATP8B4*	31.14	40.67	0	2641.01	3257.69	1979.05
novel_circ_0002934	471	Exon	*RAB11A*	2701.79	3236.86	2108.44	7786.09	7939.65	8398.71
novel_circ_0007993	752	Exon	*IL1R1*	47.19	103.71	17.57	2624.60	1723.90	2305.54
novel_circ_0008106	574	Exon	*DCUN1D2*	1758.97	1012.37	2483.11	4797.46	3663.55	6148.49
novel_circ_0011216	617	Intergenic	*–*	76.39	282.80	204.83	2545.69	2420.46	2183.22
novel_circ_0013493	1305	Exon	*MINDY4*	309.00	1073.48	2044.87	4491.23	4247.59	3589.50
novel_circ_0018397	364	Exon	*CRIM1*	659.10	643.08	921.63	4999.74	7348.63	5795.70
novel_circ_0022193	291	Intergenic	*–*	433.95	274.59	711.29	2525.95	2475.04	4148.50
novel_circ_0024784	636	Exon	*FGFR2*	642.88	508.04	1615.03	4921.98	5726.85	5453.15

*The latter six columns were referred to the Normalized TPM value. SYF, small yellow follicle; F6, smallest hierarchical follicle; F1, largest hierarchical follicle. G, granulosa cell; T, theca cell.*

**FIGURE 3:**
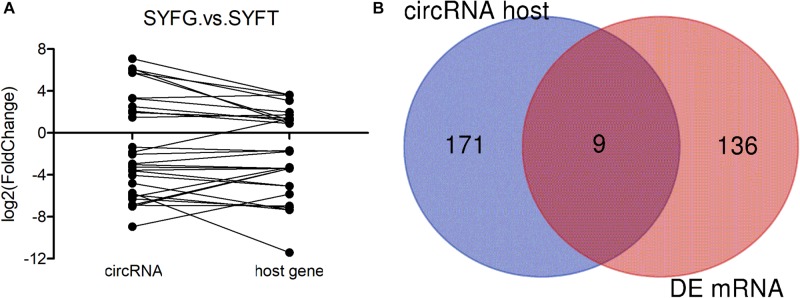
Expression level of relationship between circRNA and its host genes. **(A)** Fold change of circRNA and its host genes; **(B)** Venn diagram of DE circRNA host genes and DE mRNA in SYFG vs. SYFT comparison group. SYFG, granulosa cells of small yellow follicle; SYFT, theca cells of small yellow follicle.

Many mRNAs were differentially expressed in the SYF stage, so the relationship between circRNA and mRNA expression was investigated in terms of the fold changes and the overlap with host gene expression (Figure 3). The results showed that the fold changes in circRNA and mRNA expression were irregular, with the fold changes in the circRNAs greater, smaller, or the same as those in the mRNAs (Figure 3A). The expression of only nine genes overlapped with the expression of the host genes of the DE circRNAs and mRNAs in the SYFG vs. SYFT comparison (Figure 3B). Detailed information on the overlapping genes is given in Supplementary Table S6.

### Validation of circRNA and RNA-seq Data

**FIGURE 4:**
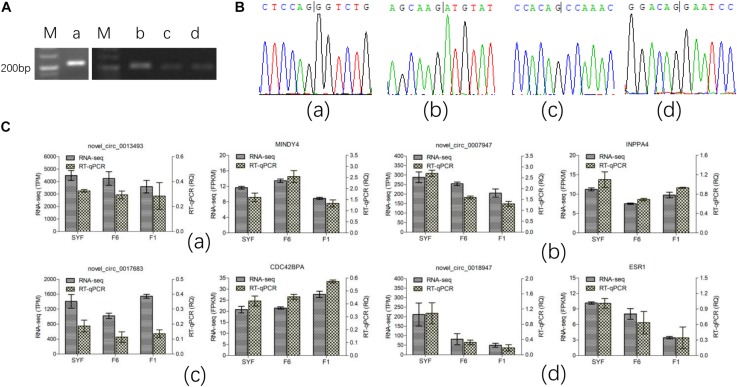
Experimental validation of circRNA. **(A)** PCR products were detected in agarose gel electrophoresis; **(B)** Sanger sequencing for the back-splice site of circRNA circulation; **(C)** RT-qPCR test for the expression level. Figures of small letter a, b, c and d represents 4 circRNAs and its host genes as following order: novel_circ_0013493(MINDY4), novel_circ_0007947(INPP4), novel_circ_0017683(CDC42BPA) and novel_circ_0018947(ESR1), respectively.

We selected four circRNAs from the nine circRNAs mentioned above to test their splice sites, the accuracy of the RNA-seq of the circRNAs, and their host genes. Divergent primers were designed to confirm the back-splice sites of the predicted circRNAs (Supplementary Table S2) and the products were detected with agarose gel electrophoresis (Figure 4A). The products of the expected sizes were sequenced with Sanger sequencing (Figure 4B), which showed that the splice sites were those predicted by the software.

Only the transcripts in TCs were validated with RT–qPCR because the volume of GCs available was limited. The results are shown in Figure 4C, and are consistent with the RNA-seq data, confirming that the results of RNA-seq were accurate.

### Bioinformatic Analysis of DE Transcripts

**FIGURE 5:**
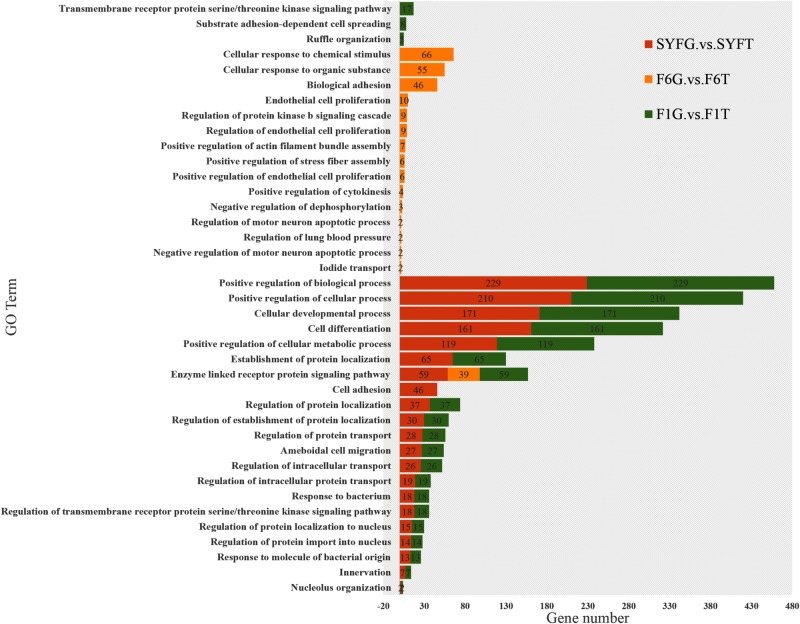
Biological process of host genes of DE circRNAs in different comparison group. DE, differentially expressed. SYF, small yellow follicle; F6, the smallest hierarchical follicle; F1, the largest hierarchical follicle. G, granulosa cell; T, theca cell.

**FIGURE 6:**
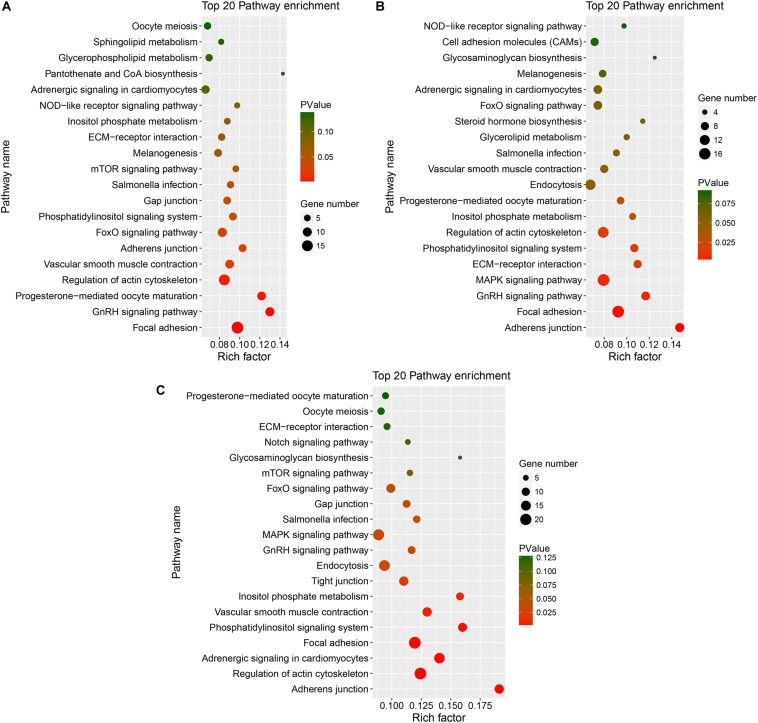
KEGG of host genes of DE circRNAs in different comparison group. **(A–C)** represents Top 20 pathways for host genes of circRNAs for SYFG vs. SYFT, F6G vs. F6T, and F1G vs. F1T comparison group, respectively. DE, differentially expressed; SYF, small yellow follicle; F6, the smallest hierarchical follicle; F1, the largest hierarchical follicle. G, granulosa cell; T, theca cell.

We performed an enrichment analysis of the host genes of the DE circRNAs to predict the functions of the circRNAs with KOBAS, and the results are shown in Figures 5, 6. Figure 5 shows that most of the same GO terms were enriched in the SYFG vs. SYFT and F1G vs. F1T comparisons, and included regulation of biological process, cellular development, cell differentiation, and cellular metabolic process. However, in the F6G vs. F6T comparison, the GO terms enriched were cellular response to chemical stimulus/organic substance, biological adhesion, biological proliferation, and endothelial cell proliferation.

Figure 6 shows the major KEGG pathways enriched in DE circRNAs in the three comparison groups. The same 12 pathways were enriched in all three groups, including the FoxO signaling pathway, GnRH signaling pathway, ECM–receptor interaction, and progesterone-mediated oocyte maturation. The NOD-like receptor signaling pathway and melanogenesis were also enriched in the SYFG vs SYFT comparison. Cell adhesion molecules and steroid hormone biosynthesis were enriched in the F6G vs. F6T comparison. The MAPK signaling pathway was enriched in the F1G vs. F1T comparison.

**FIGURE 7:**
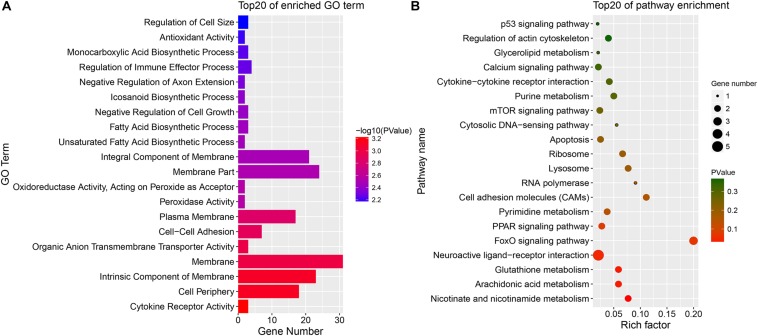
Enrichment DE mRNAs in different comparison group. **(A)** GO enrichment for DE mRNA; **(B)** KEGG enrichment for DE mRNA. SYF, small yellow follicle; F6, the smallest hierarchical follicle; F1, the largest hierarchical follicle.

Because DE mRNAs were only detected in the SYFG vs. SYFT comparison, GO and KEGG analyses were only performed for these stages, and the results are shown in Figure 7. The significant GO terms included cytokine receptor activity, cell periphery, membrane, and some fatty acid biosynthetic processes. The KEGG pathways included the FoxO signaling pathway, calcium signaling pathway, and apoptosis and some immunity-associated pathways, such as the PPAR signaling pathway and p53 signaling pathway were also enriched.

### Co-expression of Transcripts

**FIGURE 8:**
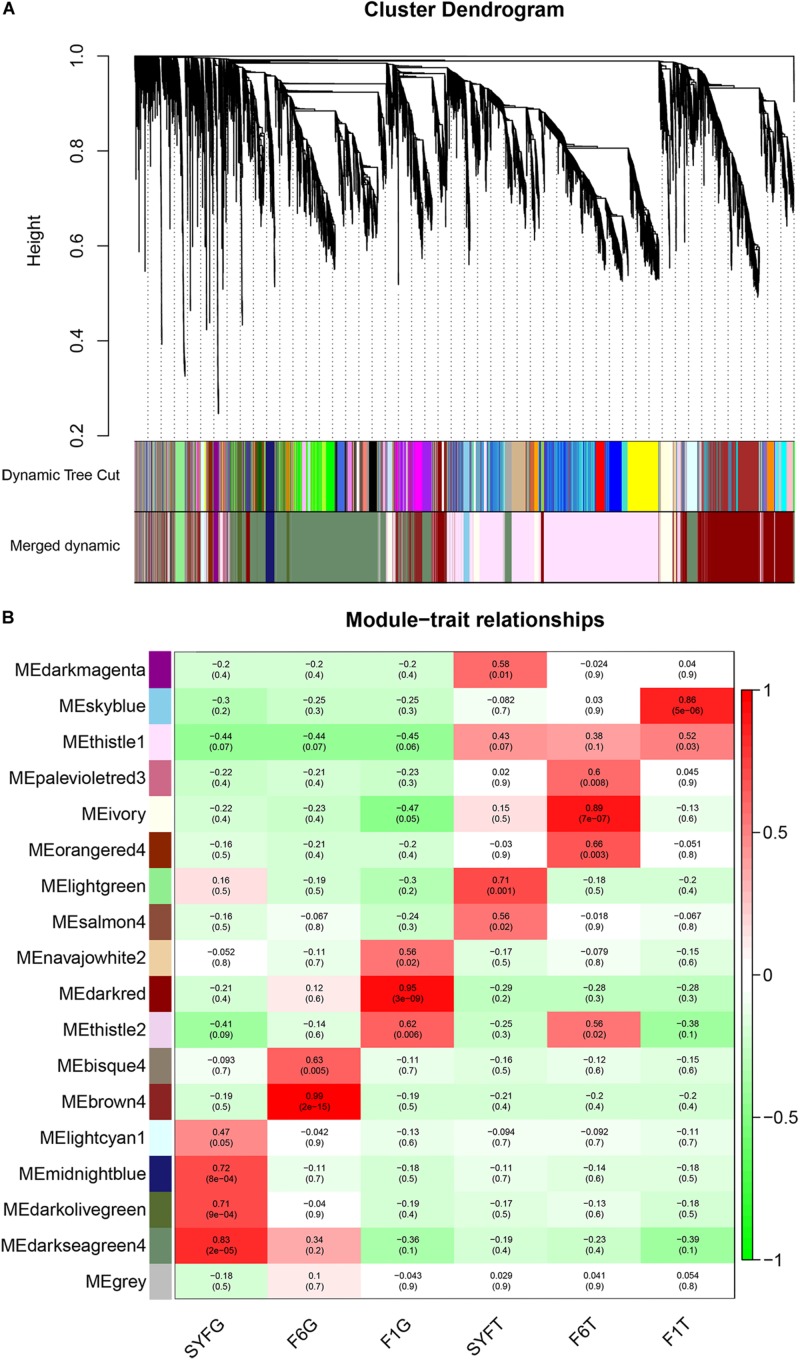
Visualization of transcripts co-expression modules and module-trait relationships. **(A)** Visualization of transcripts co-expression modules, clustering dendrogram of transcripts, with dissimilarity based on topological overlap; **(B)** Module-stage/tissue relationships and corresponding *p-*values, on the left, different colors represent different modules. Each cell contains the correlation and *p*-value given in the parentheses. Red, positive correlation; green, negative correlation; white, none correlation. SYF, small yellow follicle; F6, the smallest hierarchical follicle; F1, the largest hierarchical follicle. G, granulosa cells; T, theca cells.

To evaluate the potential functions of the circRNAs and their interactions with mRNAs, the WGCNA method was used to systematically identify the gene sets related to specific biological processes. After a series of calculations, a total of 18 modules were identified, with an average of 651.17 transcripts per module (Supplementary Table S7). The module visualization and module–trait correlations are show in Figure 8. Sixteen modules were positively associated with different follicle cells (correlation ≥ 0.5, *p* < 0.05; Figure 8B). Notably, five modules were strongly related to five follicle cells, except in the SYFT stage (correlation ≥ 0.8, *p* < 0.05; Figure 8B).

**FIGURE 9:**
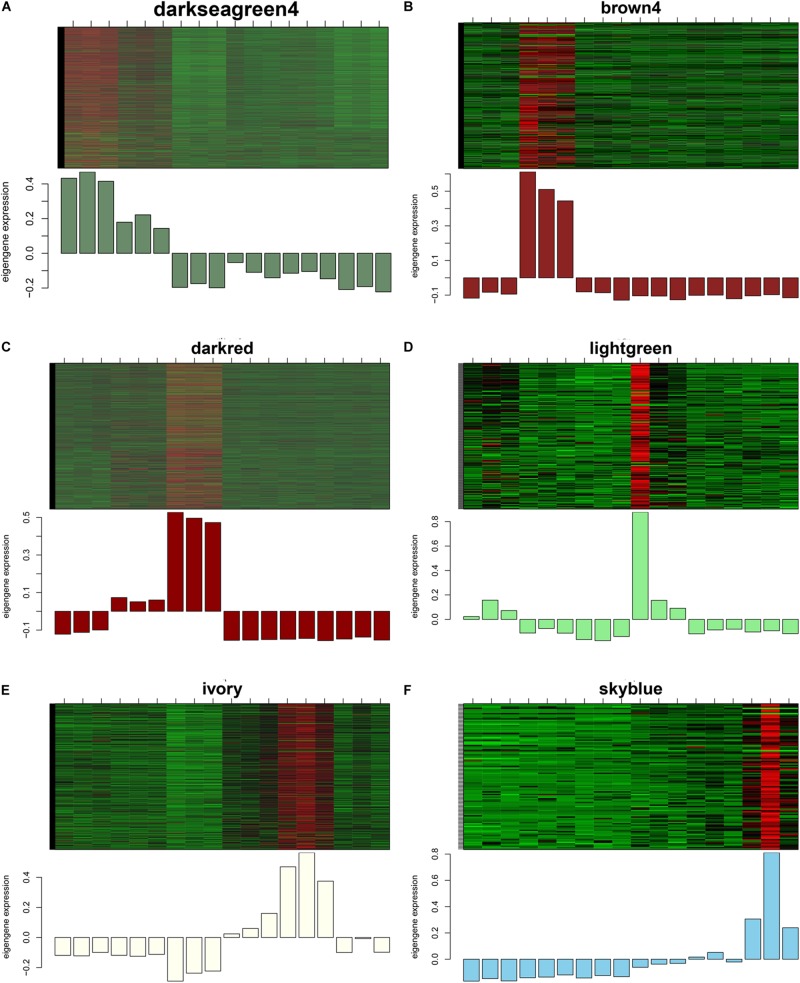
Heatmap and bar plot of expression level in six stage-specific modules. Six modules represent **(A–F)** darksegreen4, brown4, darkred, lightgreen, ivory, and skyblue with specific expression in SYFG, F6G, F1T, SYFT, F6T, and F1T, respectively. Samples were sorted in the order of: SYFG1-3, F6G1-3, F1G1-3, SYFT1-3, F6T1-3 and F1T1-3.

GS and MM were calculated for all transcripts within each module, especially those with high module–trait correlations (correlation > 0.5, *p* < 0.01), to identify the modules specific to the different stages of follicle development (Supplementary Figure S1). The correlations between GS and MM usually showed that the transcripts were strongly associated with a specific stage or tissue (correlation > 0.5, *p* < 0.01), except the thistle1 module (correlation = 0.39). The module eigengene (ME) was calculated to construct a heatmap and bar plot of the co-expression of the mRNAs and circRNAs in the different modules, and six stage-specific modules were identified (Figure 9). The transcripts in the modules darkseagreen4, darkred, lightgreen, ivory, and skyblue were highly upregulated in the SYFG, F1G, SYFT, F6T, and F1T stages, respectively, whereas the transcripts in brown4 were up-regulated in the F6G stage and down-regulated in the other modules. To simplify the downstream analysis, the six modules darkseagreen4, brown4, darkred, lightgreen, ivory, and skyblue are referred to as SYFGm, F6Gm, F1Gm, SYFTm, F6Tm, and F1Tm, respectively.

### Bioinformatic Analysis of Hub Genes

Recently, the analysis of hub genes has been recognized as a promising approach to identifying key biological process genes (Nibbe et al., 2010). In the abovementioned six modules, we considered those transcripts with GS > 0.4 and MM > 0.8 to be putative hub genes (Supplementary Table S8), and 1469, 405, 1088, 78, 378, and 81 hub transcripts were identified in the SYFGm, F6Gm, F1Gm, SYFTm, F6Tm, and F1Tm modules, respectively. We performed a GO enrichment analysis on the mRNAs and host genes of the circRNAs in each module (Table 4). The hub genes in SYFGm were mainly significantly enriched in intracellular and organelle activity including intracellular part, intracellular organelle part, and intracellular organelle. Examples of these genes include *RPL23*, *ORC5*, *HMGB3*, and *PIK3CA*. The GO terms of the hub genes in F6Gm were mainly enriched in cellular process and protein regulation process, and included *PAK1*, *HDAC2*, *SENP5*, and *TMED5*. The hub genes in F1Gm, such as *ATF2*, *SUPT3H*, and *TUBGCP2*, may be involved in mitosis and cell development. The hub genes in SYFTm, such as *SYPL1*, *SGMS2*, and *SOD1*, may be involved in germ cell development. The hub genes in F6Tm, such as *C1D*, *SOD1*, and *IPO13*, may participate in intracellular processes. KEGG pathways analyses were performed for the specific stages (Table 5). The pathways identified in different specific modules differed. The hub genes in SYFGm may be involved in the cell cycle, RNA transport, and polymerase. The hub genes in F6Gm were enriched in oxocarboxylic acid metabolism, oocyte meiosis, and the MAPK signaling pathway. The hub-gene-enriched pathways in F1Gm included endocytosis, steroid biosynthesis, and the FoxO signaling pathway. The hub-gene-enriched pathways in SYFTm included cell cycle, drug metabolism cytochrome P450, and the calcium signaling pathway; in F6Tm, they included the biosynthesis of unsaturated fatty acids, fatty acid metabolism and protein export; and in F1Tm, they included focal adhesion, vascular smooth muscle contraction, and apoptosis.

**TABLE 4:** Top 10 GO terms of hub genes in different specific modules.

	**#Term**	**rich_factor**	***P*-Value**	**Example gene**
Darkseagreen4 for SYFG	Intracellular part	0.10	6.69E-10	*EXOSC10, ODC1, NPL*
	Nucleus	0.11	1.08E-09	*RPL23, HMGB3, F6*
	Intracellular	0.10	3.84E-09	*ODC1, NPL, TESC*
	Intracellular organelle part	0.11	4.36E-09	*CCDC92, ORC5*
	Organelle part	0.11	9.83E-09	*PNPLA7, ORC5*
	Intracellular organelle	0.10	1.37E-08	*PNPLA7, HMGB3*
	Intracellular membrane-bounded organelle	0.10	2.49E-08	*WASHC1, HMGB3*
	Cytosolic ribosome	0.37	1.23E-07	*RPS4, RPS13, RPS17*
	Membrane-bounded organelle	0.10	5.04E-07	*WASHC1, RPL23, HMGB3*
	Macromolecular complex	0.11	6.18E-07	*WASHC1, PIK3CA, ORC5*
Brown4 for F6G	Cellular protein metabolic process	0.03	1.95E-05	*SENP5*
	Protein metabolic process	0.03	2.17E-05	*SENP5, PAK1*
	Protein modification process	0.03	1.15E-04	*SENP5, PAK1*
	Cellular protein modification process	0.03	1.15E-04	*BRMS1L, KCNAB1*
	Coenzyme binding	0.13	2.25E-04	*AIFM2, CRY2, BRMS1L*
	Negative regulation of macromolecule metabolic process	0.04	2.25E-04	*P3H1, UBAC2, FBXO18*
	Regulation of ERAD pathway	0.60	2.33E-04	*UBAC2, EDEM2, HDAC2*
	Regulation of protein metabolic process	0.04	2.63E-04	*PAK1, USP25, SENP5*
	Macromolecule modification	0.03	2.68E-04	*BRMS1L, PAK1, NDFIP2*
	Endoplasmic reticulum	0.05	3.10E-04	*TMED5, P3H1, EDEM2*
Darkred for F1G	Clathrin binding	0.22	3.15E-03	
	Clathrin-dependent endocytosis	0.20	3.77E-03	
	Clathrin-coated pit	0.18	4.43E-03	
	Microtubule cytoskeleton organization involved in mitosis	0.17	5.14E-03	*ARHGEF10, TUBGCP2*
	Mitotic spindle assembly	0.17	5.14E-03	*ARHGEF10, TUBGCP2*
	Positive regulation of cell development	0.04	8.21E-03	*PLPPR5, SEMA3A*
	Peptide-lysine-*N*-acetyltransferase activity	0.13	8.47E-03	*ATF2, SUPT3H*
	Centrosome duplication	0.13	8.47E-03	*ARHGEF10, TUBGCP2*
	Histone acetyltransferase activity	0.13	8.47E-03	*ATF2, SUPT3H*
	Peptide *N*-acetyltransferase activity	0.12	9.42E-03	
Lightgreen for SYFT	Membrane depolarization during action potential	0.25	6.33E-04	
	Membrane depolarization	0.13	1.88E-03	
	Action potential	0.10	3.16E-03	*TPCN3, NALCN*
	Oogenesis	0.10	3.16E-03	
	Female gamete generation	0.10	3.45E-03	
	Integral component of organelle membrane	0.05	1.12E-02	*SYPL1, SGMS2*
	Intrinsic component of organelle membrane	0.05	1.17E-02	*SYPL1, SGMS2*
	Voltage-gated ion channel activity	0.05	1.28E-02	*TPCN3, NALCN*
	Voltage-gated channel activity	0.05	1.28E-02	*TPCN3, NALCN*
	Germ cell development	0.05	1.28E-02	*SOD1*
Ivory for F6T	Intracellular part	0.03	7.31E-10	*C1D, CMC1, C1D*
	Macromolecular complex	0.04	8.74E-10	*DDX1, MCM6, MZT1*
	Intracellular organelle part	0.04	1.27E-09	*NME7, IPO13, MZT1*
	Intracellular organelle	0.03	2.09E-09	*SOD1*
	Intracellular	0.03	3.23E-09	*C1D*
	Organelle part	0.04	3.29E-09	*NME7, IPO13, MZT1*
	Intracellular membrane-bounded organelle	0.03	4.32E-09	*DDX1, CMC1, HIST1H4J*
	Intracellular non-membrane-bounded organelle	0.05	4.64E-09	*C1D, ACTB*
	Non-membrane-bounded organelle	0.05	4.64E-09	*NME7, MZT1*
	Nucleus	0.04	4.76E-09	*PSMB3, DDX1, IPO13*
Skyblue for F1T	Actin binding	0.14	1.69E-09	*AFAP1, CORO1C, PLS3*
	Cytoskeletal protein binding	0.08	7.84E-09	*HSPB7, AFAP1, CORO1C*
	Actin cytoskeleton	0.12	1.16E-08	*HSPB7, ACTC1, MYLK*
	Cytoskeleton	0.05	4.99E-08	*CORO1C*
	Tissue development	0.05	3.48E-07	*MYLK, CORO1C, ITGB1*
	Adherens junction	0.09	3.70E-07	*SYNM, ACTB, ITGB1*
	Anchoring junction	0.09	4.00E-07	*ACTC1, SYNM, ACTB*
	Actin filament-based process	0.08	4.80E-07	*ACTC1, CORO1C*
	Vesicle	0.04	5.83E-07	*HPCAL1, TWSG1, THBS1*
	Protein binding	0.03	1.47E-06	*CORO1C, HPCAL1, EPB41*

*SYF, small yellow follicle; F6, smallest hierarchical follicle; F1, largest hierarchical follicle. G, granulosa cell; T, theca cell.*

**TABLE 5:** Top 10 KEGG pathways of hub genes in different specific modules.

	**#Term**	**rich_factor**	***P*-value**	**Example gene**
Darkseagreen4 for SYFG	Ribosome	0.27	1.38E-07	*RPL24, RPS17*
	Cell cycle	0.22	1.15E-05	*CCNE1, ORC5*
	mRNA surveillance pathway	0.24	1.77E-04	*RNMT, EIF4A3*
	RNA transport	0.17	1.02E-03	*EIF2B5*
	Lysosome	0.18	1.42E-03	*ATP6AP1, GNPTG, ENTPD4*
	Lysine degradation	0.24	3.33E-03	*SUV39H2, NSD3, NSD2*
	DNA replication	0.25	8.18E-03	*MCM3, DNA2, RFC3*
	Glycerophospholipid metabolism	0.16	2.06E-02	*PGS1, AGPAT4, AGPAT5*
	RNA polymerase	0.25	2.43E-02	*POLR2B, POLR3E*
	Ribosome biogenesis in eukaryotes	0.16	3.07E-02	*UTP18, LSG1, UTP6*
Brown4 for F6G	Oxocarboxylic acid metabolism	0.17	2.16E-02	*BCAT1, ACO2*
	Inositol phosphate metabolism	0.07	2.22E-02	*PIKFYVE, PLCD4*
	Spliceosome	0.05	2.48E-02	*PRPF40A, PRPF8, THOC2*
	Pantothenate and CoA biosynthesis	0.14	2.79E-02	*BCAT1*
	Phosphatidylinositol signaling system	0.05	6.14E-02	*PIKFYVE, PLCD4*
	Oocyte meiosis	0.05	6.14E-02	*CDC26, PPP1CC, FBXW11*
	Biosynthesis of amino acids	0.06	6.21E-02	*BCAT1, MAT2B, ACO2*
	Protein processing in endoplasmic reticulum	0.04	6.73E-02	*UGGT1, EDEM1, SEC24D*
	Citrate cycle (TCA cycle)	0.08	7.25E-02	*ACO2, DLAT*
	MAPK signaling pathway	0.03	8.75E-02	*PAK2, NFATC3*
Darkred for F1G	Endocytosis	0.03	2.19E-02	*SMAP1, PARD3*
	Fanconi anemia pathway	0.05	4.04E-02	*POLH*
	Glycosphingolipid biosynthesis	0.08	7.52E-02	*SialT2*
	Inositol phosphate metabolism	0.03	8.64E-02	*ITPKA*
	Steroid biosynthesis	0.07	1.18E-01	*FDFT1*
	Phosphatidylinositol signaling system	0.02	1.50E-01	*ITPKA*
	Dorsoventral axis formation	0.05	1.58E-01	*CPEB2*
	Lysosome	0.02	1.75E-01	*AP1S2*
	Tight junction	0.02	1.97E-01	*PRKCD, PARD3*
	FoxO signaling pathway	0.02	2.14E-01	*PRKAG2*
Lightgreen for SYFT	Glycosphingolipid biosynthesis	0.07	5.59E-02	*ST3GAL6*
	Cell cycle	0.02	6.11E-02	*WEE2*
	Drug metabolism cytochrome P450	0.04	9.85E-02	
	Nicotinate and nicotinamide metabolism	0.04	9.85E-02	*NMNAT3*
	Metabolism of xenobiotics by cytochrome P450	0.04	1.02E-01	
	Calcium signaling pathway	0.01	1.05E-01	
	Glutathione metabolism	0.03	1.26E-01	
	Sphingolipid metabolism	0.03	1.36E-01	*SGMS2*
	Amino sugar and nucleotide sugar metabolism	0.02	1.49E-01	*GNPDA2*
	RNA degradation	0.02	2.09E-01	*BTG4*
Ivory for F6T	Proteasome	0.29	1.33E-08	*PSMB3, PSMB2, PSMC3*
	Glutathione metabolism	0.12	3.42E-03	*DNTTIP2*
	Biosynthesis of unsaturated fatty acids	0.17	4.72E-03	*HACD3*
	Ribosome	0.06	6.78E-03	*RPL24, MRPS11, RPL9*
	Cell cycle	0.06	9.28E-03	*CDKN1B, MCM6*
	RNA degradation	0.07	2.11E-02	*EXOSC8, LSM7, CNOT7*
	Spliceosome	0.05	2.48E-02	*SNRPB2, SNRPG, LSM7*
	Fatty acid metabolism	0.08	3.17E-02	*HACD3*
	Phagosome	0.04	4.42E-02	*STX18, SEC61B, DYNC1I2*
	Protein export	0.11	4.63E-02	*SEC61B, SRP72*
Skyblue for F1T	Focal adhesion	0.09	1.78E-08	*MYLK, ITGA8*
	Regulation of actin cytoskeleton	0.08	4.19E-07	*MYLK, ROCK2*
	ECM receptor interaction	0.13	3.65E-06	*COL4A2, ITGA8*
	Cardiac muscle contraction	0.11	5.28E-04	*ACTC1, TPM1, TPM3*
	Adrenergic signaling in cardiomyocytes	0.06	1.03E-03	*ACTC1, TPM1, MAPK1*
	Vascular smooth muscle contraction	0.07	1.30E-03	*MYLK, MRVI1, ROCK2*
	Phagosome	0.05	5.06E-03	*ACTB, ITGB1*
	Apoptosis	0.05	1.39E-02	*FAS, CTSS, MAPK1*
	Cell adhesion molecules (CAMs)	0.05	3.04E-02	*ITGB1, ITGA8, ITGAV*
	Oocyte meiosis	0.05	3.26E-02	*MAPK1, FBXW11*

*SYF, small yellow follicle; F6, smallest hierarchical follicle; F1, largest hierarchical follicle. G, granulosa cell; T, theca cell.*

**FIGURE 10:**
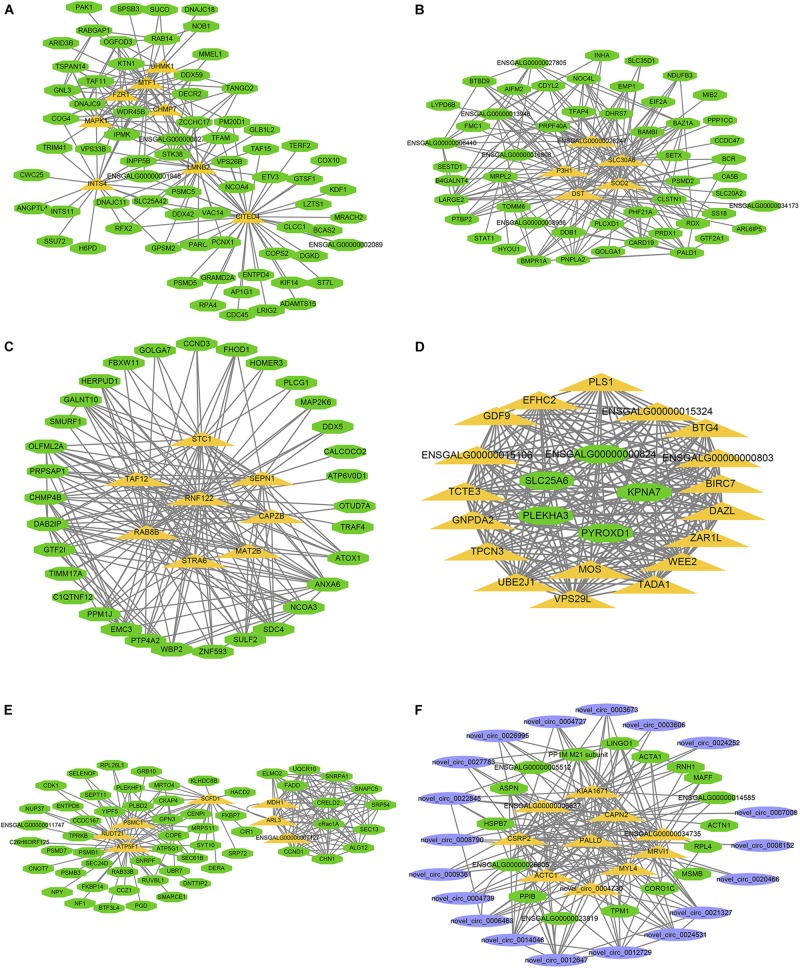
Visualization of hub genes in specific module. Network of **(A–F)** represent top hub genes in module darksegreen4, brown4, darkred, lightgreen, ivory, and skyblue, respectively. Green nodes represent protein-coding gene, blue nodes represent circRNA, orange nodes represent highly connected intramodular hub genes with edge number ≥ 15.

The larger the weighted value, the stronger the proof that a gene is a trait-associated hub gene. Therefore, networks of the top 200 edges ranked with weighted values were visualized with Cytoscape (Figure 10). In the network, the central hub genes with the edge numbers ≥ 15 were considered to be highly connected intramodular hub genes (designated ‘central hub genes’). In SYFGm, six central hub genes were identified: *UHMK1*, *FZR1*, *MAPK1*, *INTS4*, *LMNB2*, and *CITED4*. In F6Gm, five central hub genes were identified: *ENSGALG00000028247*, *P3H1*, *SLC30A6*, *DST*, and *SOD2*. In F1Gm, eight central hub genes were identified: *SCT1*, *TAF12*, *SEPN1*, *RNF122*, *RAB8B*, *CAPZB*, *STRA6*, and *MAT2B*. In SYFTm, more than 10 central hub genes were identified, including *GDF9*, *BTG4*, *TCTE3*, *BIRC7*, and *PLS1*. In F6Tm, seven central hub genes were identified: *ATP5F1*, *NUDT21*, *PSMC1*, *SCFD1*, *MDH1*, *ARL3*, and *ENSGALG00000007127*. In F1Tm, one circRNA (*novel_circ_0004730*) and nine protein-coding genes (*CAPN2*, *PALLD*, *MYL4*, *ACTC1*, *MRVI1*, etc.) were identified.

## Discussion

Follicle development is a correctly ordered series of biological processes, including follicle recruitment, follicle selection, and ovulation, and plays a key role in the laying performance of hens, which is a major research hotspot for poultry breeders (Stephens and Johnson, 2016, 2017; Peng et al., 2018). Follicle cells mainly include three cell types, GCs in the granulosa layer, TCs in the theca layer, and oocyte, and each cell type has specific functions in follicle development. To provide a comprehensive view of the transcriptomes during GC and TC growth in the follicles of chickens, including both circRNAs and protein-coding genes, whole-transcriptome analyses were performed to identify the genes involved and their regulatory roles.

circRNAs are a novel type of non-coding RNA that plays various roles in life events. To our knowledge, this is the first study of the circRNAs involved in chicken follicle development in terms of GCs and TCs, although systematic searches for chicken circRNAs have been conducted in the liver (Zhang et al., 2017) and embryonic muscle (Ouyang et al., 2017). In this study, we identified over ten thousand circRNAs in GCs and TCs, 8127 of which were abundant in both cell types, and found that they share similar features in GCs and TCs. In total, 23,769 mRNAs were detected in GCs and TCs. Notably, more circRNAs and mRNAs were detected in TCs than in GCs, consistent with a previous study (Jing et al., 2018). This suggests that transcripts are preferentially expressed in TCs because of the complexity of the thecal layer, which contains aromatase cells, fibroblast, and interstitial cells (Bahr, 1991), whereas the components of the granulosa layer are much simpler (Johnson, 2015). A DE analysis of these circRNAs and mRNAs showed that circRNAs are preferentially expressed in a tissue-specific manner, suggesting that the regulatory roles played by non-protein-coding genes at the transcription level differ from those played by protein-coding genes.

Previous reports have shown that circRNA biogenesis sometimes competes with pre-mRNA splicing (Ashwal-Fluss et al., 2014), and that some circRNAs do not colocalize with their host genes (You et al., 2015). In this study, we found that circRNA expression levels were independent of the abundance of the linear RNA isoform and that only nine genes that overlapped at the circRNA and mRNA levels were differentially expressed in the SYFG vs. SYFT comparison. A GO analysis of DE circRNAs showed that most genes differentially expressed in the SYFG vs. SYFT and F1G vs. F1T comparisons were associated with cell differentiation, cell development, and metabolic process, suggesting that the same biological events occur in the SYF and F1 follicles. The GO terms enriched in the F6G vs. F1T comparison differed from those for the other two comparisons, and were enriched in cellular response, cellular response, and endothelial cell proliferation. A possible explanation is that the processes in each stage of follicular cell development in the ovary differ, and that the SYF and F1 follicles undergo selection and ovulation, whereas in the F6 stage, GCs and TCs proliferate.

Most KEGG pathways identified in the three comparisons were related to some reproduction trait, such as the FoxO signaling pathway, GnRH signaling pathway, and progesterone-mediated oocyte maturation, which have been reported to play key roles in follicular development (Peng et al., 2018). In the enrichment analysis of DE mRNAs, only the mRNAs differentially expressed in the SYFG vs. SYFT comparison were enhanced in some fatty acid biosynthetic process and reproduction pathways. SYF is the main stage in which estrogen is produced (Bahr, 1991), and fatty acid biosynthetic processes provide the components of estrogen. The differences detected in the enrichment analyses of circRNAs and mRNAs may imply their different regulatory roles at the transcription level, insofar as circRNAs may function in cell development whereas mRNA may play a key role in physiological functions.

Remarkably, most of the highly expressed circRNAs were derived from exons in both GCs and TCs, as shown in Tables 2, 3 but only about 40% of the circRNAs identified in the RNA-seq data were produced from exons (Supplementary Table S4). The host genes of the DE circRNAs that were most strongly differentially expressed in the GCs and TCs in chickens in this study also produce a series of isoforms as circRNAs in humans (Salzman et al., 2013; Rybak-Wolf et al., 2015), which have been published in circBase (Glazar et al., 2014) (except *YBX3*, *EPT1L*, and *MINDY4*). To date, no research has examined the functions of these circRNAs *in vitro* or *in vivo*, but isoforms of the mRNAs transcribed from the host genes of these circRNAs play a regulatory role in mammalian reproduction. Insulin-like growth factor receptor (IGF1R) is a functional receptor for IGF1 and IGF2, which has been shown to promote the nuclear maturation of oocytes and to stimulate cell proliferation and inhibit apoptosis (Sirotkin, 2011). *ESR1* encodes one of the estrogen receptors that mediate the regulation of mammalian reproduction by the hormone estradiol (Edson et al., 2009). Interleukin 1 receptor (*IL1R1*) increases the expression of the FSH receptor (*FSHR*) in mouse primary GCs (Uri-Belapolsky et al., 2017). However, there is little information on the functions of these genes in the chicken follicle, in which GCs and TCs have different functions from those in mammalian ovaries (Johnson, 2015). Interestingly, *novel_circ_0002934* produced from the *RAB11A* gene was highly expressed in both cell types. A previous study reported that RAB11A may act as a major regulator of membrane delivery during cytokinesis (Chu et al., 2009) and plays a supporting role in autophagy (Huttenhower et al., 2009). The regression of the chicken postovulatory follicle is mediated by autophagy (Lin et al., 2018), and autophagy signaling pathways are critical in controlling broodiness in the goose (Yu et al., 2016). During the follicular growth process, most SYF follicles undergo atresia (Hocking, 2009), which involves a complex array of mechanisms that modulate GC and TC apoptosis (Johnson, 2003; Lebedev et al., 2006; Kim et al., 2016). However, the effect of autophagy on follicle atresia before follicle selection has received little attention. Therefore, research in autophagy during follicle development requires further investigation with reference to the *RAB11A* gene and its circular RNA.

Functional studies of circRNAs usually focus on their microRNA (miRNA) sponges (Fu and Jiang, 2018), and research on the expression networks of circRNAs and protein-coding genes has been limited. In this study, we used WGCNA (Langfelder and Horvath, 2008) to construct specific modules and networks of interactions during follicle development. According to the expression profiles of these modules, we finally identified six specific functional modules and thousands of hub genes that specific positively correlate with the six follicular tissues. Expression level of the darkseagreen4, brown4, darkred, lightgreen, ivory, and skyblue modules peaked at SYFG, F6G, F1G, SYFT, F6T, and F1T, respectively. Consequently, we explored enrichment analysis in the six specific modules. The genes present in a single module have a common function and affect tissue- or stage-specific biological processes. Enrichment analyses of the hub genes in the different modules showed that different terms and pathways were enriched in the different modules. SYFGs are undifferentiated and steroidogenically inactive (Tilly et al., 1991a), while SYFTs is the stage at which the differentiation of the internal and external thecal layers occurs. Apoptotic DNA cleavage was detected in GCs and TCs (Tilly et al., 1991b), and the majority of SYFs undergo atresia, with only one SYF growing to the hierarchical follicle each day in a clutch, which suggests that SYF is a complex tissue, involving multiple biological processes, including growth and apoptosis (Tilly et al., 1991b). To understand the fine mechanisms operating in this special stage, more studies are required of differently sized follicles in the SYF pool, using new technologies, such as single-cell sequencing, a powerful new technology for studying rare cells (Picelli, 2017).

Another module enrichment analysis may identify the main functions of different tissues, e.g., the regulation of the endoplasmic-reticulum-associated protein degradation (ERAD) pathway (which had a high enrichment factor of 0.6 in F6Gm) and oxocarboxylic acid metabolism (with an enrichment factor of 0.17). The ERAD and oxocarboxylic acid pathways have been shown to act in cell proliferation (McKeehan and McKeehan, 1979; Diaferia et al., 2013). The proliferation of GCs and TCs in F6 follicles is the beginning of a rapidly growing hierarchy (Dunn et al., 2003). The genes in the F6 module may participate in the proliferation of the follicle. The hub genes in F1Gm were enriched in clathrin binding, endocytosis, steroid biosynthesis, and so on, and the hub genes in F1Tm were enriched in actin binding, focal adhesion, vascular smooth muscle contraction, and so on. The F1 follicle is the main site of progesterone production (Lee et al., 1998). The reduction in tight junctions in the follicular theca is the key factor affecting ovulation (Lebedev et al., 2006). From our WGCNA analysis and the different functions of GCs and TCs, we draw the conclusion that in the different stages of follicle development, the cells undertake different biological functions, for which different genes are required.

As hub genes are principle regulators in modules, we designated the genes with ≥15 edge numbers connectivity as central hubs in each network. Clearly, most hub components in specific modules are different from each other. The central hub genes in specific modules, such as *MAPK1* in SYFGm (Woods et al., 2009), *SOD*2 in F6Gm (Jia et al., 2016), and *GDF9* in SYFTm (Johnson, 2012), have been described in previous reports, but most of them were first reported in our study. *CITED4* was one of the very central hub genes in SYFGm, and CITED4 targets luteinizing hormone (LH) and ERK1/2, triggering ovulation in mice (Zhang et al., 2014). Before follicle selection, the LH receptor (LHR) is present at low levels in prehierarchical follicles (Johnson et al., 2004), and GCs preferentially express LHR compared with TCs (Yao and Bahr, 2001). Therefore, we hypothesized that *CITED4* is involved in the regulation of LHR expression in SYFGs, thus mediating follicle selection. *GDF9*, *MOS*, *BTG4*, and another 15 genes were the central hub genes in SYFTm. GDF9 increased the number of human ovary TCs in culture, but inhibited thecal steroidogenesis (Yamamoto et al., 2002). *MOS* is differentially expressed in the ovarian follicles of Bashang long tail chickens and Hy-line brown layers, and its expression may be regulated by several of long non-coding RNAs (lncRNAs) (Peng et al., 2018). *BTG4* has antiproliferative properties and is a novel tumor suppressor (Toyota et al., 2008). It is speculated that these genes and other genes or transcripts in SYFTm are involved in a large network that regulates thecal growth and its functions.

*SOD2*, a central hub gene in F6Gm, is antioxidant activity-related genes playing an important role in inhibiting ROS biosynthesis. It was previously reported that SOD2 involved in pharmacological protection of the mouse ovaries (Qin et al., 2018) and had a role in goat granulosa cells proliferation (Yao et al., 2017). Given its roles in antioxidation and proliferation, *SOD2* is likely a potential key gene modulating granulosa cell proliferation and protection in F6G stage. *STC1*, a central hub gene in F1Gm, may be involved in human ovarian tumorigenesis via promoting proliferation and inhibiting apoptosis of tumor cells (Liu et al., 2010). In a granulosa cells research, *SCT1* had been proved to dampen the gonadotropin stimulation of granulosa cell differentiation by paracrine regulation and inhibit progesterone secretion (Luo et al., 2004). It is tempting to speculate that *STC1* may have a pivotal role in the F1G stage. *MDH1*, a central hub gene in F6Tm, is a glucose metabolism-related enzyme gene may have an important role of the activation and maintenance of the mouse ovarian follicular fool (Sutherland et al., 2012). A study from Seol et al. (2006) showed that genes involved in glucose utilization ensure chickens have a rapid follicle development from F6 to F1 follicle, even though glycolysis and β-oxidation are not modulated by follicle growth. F6 stage is the begining of hierarchal follile phase which suggested that *MDH1* may be involved in energy utilization to participate in rapid follicle growth. *CAPN2*, a central hub gene in F1Tm, was implicated to important cellular process including apoptosis and survival (Storr et al., 2012), and had been reported to be upregulated by hCG in bovin granulosa cells of ovulatory follicle and may contribute to ovulation processes (Lussier et al., 2017). Thus we speculated that *CAPN2* may have a similar function on the F1T cells in the ovulation. Despite these candidate hub genes findings, questions remain. Further functional characterization of these hub genes in the future will shed light on how they interact with each other to modulate follicle development.

It is noteworthy that we only detected one very top hub circRNA, *novel_circ_0004730*, in F1Tm. This may be because only a few circRNAs function at the transcription level, and that most of them are involved in crosstalk (Tay et al., 2014). *novel_circ_0004730* is produced from the *SAR1B* gene (Supplementary Table S4), which regulates cholesterol homeostasis (Sane et al., 2015). Previous studies have mainly addressed the function of SAR1B in lipid secretion and cholesterol biosynthesis (Fryer et al., 2014), and there is little information on the role of this gene in reproduction, especially in follicle development.

An intriguing observation is that *ESR1* was differentially expressed as circRNA and mRNA isoforms in the SYFG vs. SYFT comparison, and was one of the hub genes in F1Gm, with GS = 0.96 and MM = 0.89. The *ESR1* gene is mainly expressed in the theca in the mouse ovary (Britt and Findlay, 2003), and is one of the estrogen receptors that mediate the regulation of mammalian reproduction by estradiol through autocrine and paracrine mechanisms (Edson et al., 2009). It has also been suggested that estrogen is required to maintain the integrity of the follicle wall (Bahr, 1991). A previous study showed that the expression of *CYP19* was strongly associated with *ESR1* expression in ewe large follicles (Foroughinia et al., 2017), and *CYP19A1* was a hub gene in F6Tm in this study (Supplementary Table S8). Estrogen is involved in both positive and negative feedback in the hypothalamic–pituitary–gonadal axis (Hocking, 2009). *ESR1* localized in SYF tissues may increase their estrogen secretion by positive feedback to estrogen, because about 50% of estrogen is produced from small follicles (Bahr, 1991), and the estrogen in F1 follicle tissues is reduced by negative feedback to estrogen (Johnson et al., 1996). The mechanism of follicular development is more complex than a simple molecular interaction. Our results indicate that *ESR1* may be a key regulator of the development of the granulosa and thecal layers and play a role in the interactions between them. Further investigation of the *ESR1* gene as the source of circRNAs and mRNAs involved in GC and TC development in chickens is required.

## Conclusion

Ovarian follicular development requires a continuous supply of endocrine, paracrine, and autocrine factors and these factors affect cell differentiation, proliferation, and apoptosis. This study extends our understanding of the circRNAs and mRNAs that affect follicle development. The numbers of circRNAs and mRNAs and the levels of their differential expression in GCs and TCs differ at different stages, suggesting that these transcripts or genes have specific functions in cells or tissues. In a WGCNA analysis, we identified six specific modules that are associated with different follicular cells or stages of follicle development. Based on the WGCNA regulatory network, we conclude that the *MAPK1* and *CITED4* in the SYFGm, *SOD2* in the F6Gm, *STC1* in the F1Gm, *GDF9* and *MOS* in the SYFTm, *MDH1* in the F6Tm, *CAPN2* and *novel_circ_0004730* in the F1Tm may participate the specific function of follicle development. Moreover, another interesting gene *ESR1*, detected by both DE analysis and WGCNA method, may be an important gene involved in follicle development, in both GCs and TCs, and is expressed as both circRNA and mRNA isoforms. This study lays a preliminary foundation for research into circRNAs and mRNAs in the development of follicles. However, the mechanisms of their actions remain to be clarified.

## Data Availability Statement

The raw data were submitted to the National Center for Biotechnology Information (NCBI) under BioProject accession numbers PRJNA481176 and PRJNA511712.

## Ethics Statement

The animal study was reviewed and approved by Animal Care Committee of Yangzhou University.

## Author Contributions

MS, JW, and GZ designed the work and wrote the manuscript. MS, TL, and FC performed the transcriptome data and prepared the figures. PW, YW, and KX collected the samples. All authors read and approved the final manuscript.

## Conflict of Interest

The authors declare that the research was conducted in the absence of any commercial or financial relationships that could be construed as a potential conflict of interest.

## Supplementary Material

FIGURE S1 Visualization of GS vs. MM. The scatter plots display the distribution of GS and MM of transcripts in stage-specific modules related to individual sample.

TABLE S1 Soft power estimated according to TOM.

TABLE S2 Primers for the validation of circRNAs or RT-qPCR.

TABLE S3 Quality control results for circRNAs and mRNAs.

TABLE S4 Novel circRNAs detected in follicles. The overlapped circRNAs in GCs and TCs were marked with red font.

TABLE S5 Feature of transcripts detected in current study.

TABLE S6 Detail information of nine overlapped genes.

TABLE S7 Modules and genes number identified in WGCNA analysis.

TABLE S8 Transcripts information in different specific modules.

## References

[B1] Alvaro Mercadal B. Imbert R. Demeestere I. Gervy C. De Leener A. Englert Y. (2015). AMH mutations with reduced in vitro bioactivity are related to premature ovarian insufficiency. *Hum. Reprod.* 30 1196–1202. 10.1093/humrep/dev042 25750103

[B2] Ashwal-Fluss R. Meyer M. Pamudurti N. R. Ivanov A. Bartok O. Hanan M. (2014). circRNA biogenesis competes with pre-mRNA splicing. *Mol. Cell* 56 55–66. 10.1016/j.molcel.2014.08.019 25242144

[B3] Bahr J. M. (1991). The chicken ovary as a model of follicular development. *Semin. Reprod. Med.* 9 352–359.

[B4] Britt K. L. Findlay J. K. (2003). Regulation of the phenotype of ovarian somatic cells by estrogen. *Mol. Cell. Endocrinol.* 202 11–17. 10.1016/s0303-7207(03)00055-8 12770724

[B5] Cai H. Li Y. Li H. Niringiyumukiza J. D. Zhang M. Chen L. (2018). Identification and characterization of human ovary-derived circular RNAs and their potential roles in ovarian aging. *Aging* 10 2511–2534. 10.18632/aging.101565 30260796 PMC6188495

[B6] Chen B. Yu J. Guo L. Byers M. S. Wang Z. Chen X. (2019). Circular RNA circHIPK3 promotes the proliferation and differentiation of chicken myoblast cells by sponging miR-30a-3p. *Cells* 8:177 10.3390/cells8020177 PMC6406597 30791438

[B7] Cheng J. Huang J. Yuan S. Zhou S. Yan W. Shen W. (2017). Circular RNA expression profiling of human granulosa cells during maternal aging reveals novel transcripts associated with assisted reproductive technology outcomes. *PLoS One* 12:e0177888 10.1371/journal.pone.0177888 PMC5482436 28644873

[B8] Chu B. B. Ge L. Xie C. Zhao Y. Miao H. H. Wang J. (2009). Requirement of myosin Vb.R*ab*11a.Rab11-FIP2 complex in cholesterol-regulated translocation of NPC1L1 to the cell surface. *J. Biol. Chem.* 284 22481–22490. 10.1074/jbc.M109.034355 19542231 PMC2755969

[B9] Diaferia G. R. Cirulli V. Biunno I. (2013). SEL1L regulates adhesion, proliferation and secretion of insulin by affecting integrin signaling. *PLoS One* 8:e79458 10.1371/journal.pone.0079458 PMC3854660 24324549

[B10] Donadeu F. X. Fahiminiya S. Esteves C. L. Nadaf J. Miedzinska K. McNeilly A. S. (2014). Transcriptome profiling of granulosa and theca cells during dominant follicle development in the horse. *Biol. Reprod.* 91:111 10.1095/biolreprod.114.118943 25253738

[B11] Dunn I. Miao Y. Morris A. Romanov M. Wilson P. Waddington D. (2003). A study of association between genetic markers in candidate genes and reproductive traits in one generation of a commercial broiler breeder hen population. *Heredity* 92 128–134. 10.1038/sj.hdy.6800396 14679392

[B12] Edson M. A. Nagaraja A. K. Matzuk M. M. (2009). The mammalian ovary from genesis to revelation. *Endocr. Rev.* 30 624–712. 10.1210/er.2009-0012 19776209 PMC2761115

[B13] Elis S. Dupont J. Couty I. Persani L. Govoroun M. Blesbois E. (2007). Expression and biological effects of bone morphogenetic protein-15 in the hen ovary. *J. Endocrinol.* 194 485–497. 10.1677/JOE-07-0143 17761888

[B14] Eresheim C. Leeb C. Buchegger P. Nimpf J. (2014). Signaling by the extracellular matrix protein Reelin promotes granulosa cell proliferation in the chicken follicle. *J. Biol. Chem.* 289 10182–10191. 10.1074/jbc.M113.533489 24573679 PMC3974987

[B15] Foroughinia G. Fazileh A. Eghbalsaied S. (2017). Expression of genes involved in BMP and estrogen signaling and AMPK production can be important factors affecting total number of antral follicles in ewes. *Theriogenology* 91 36–43. 10.1016/j.theriogenology.2016.12.023 28215684

[B16] Fryer L. G. Jones B. Duncan E. J. Hutchison C. E. Ozkan T. Williams P. A. (2014). The endoplasmic reticulum coat protein II transport machinery coordinates cellular lipid secretion and cholesterol biosynthesis. *J. Biol. Chem.* 289 4244–4261. 10.1074/jbc.M113.479980 24338480 PMC3924288

[B17] Fu Y. Jiang H. (2018). Genome-wide analysis of circular RNAs in bovine cumulus cells treated with BMP15 and GDF9. *Sci. Rep.* 8:7944 10.1038/s41598-018-26157-2 PMC5962577 29786687

[B18] Gao Y. Zhang J. Zhao F. (2017). Circular RNA identification based on multiple seed matching. *Brief Bioinform.* 19 803–810. 10.1093/bib/bbx014 28334140

[B19] Gilbert A. B. Evans A. J. Perry M. M. Davidson M. H. (1977). A method for separating the granulosa cells, the basal lamina and the theca of the preovulatory ovarian follicle of the domestic fowl (*Gallus domesticus*). *J. Reprod. Fertil.* 50 179–181. 10.1530/jrf.0.0500179 864645

[B20] Glazar P. Papavasileiou P. Rajewsky N. (2014). circBase: a database for circular RNAs. *RNA* 20 1666–1670. 10.1261/rna.043687.113 25234927 PMC4201819

[B21] Guo T. Huang L. Yao W. Du X. Li Q. Ma M. (2019). The potential biological functions of circRNAs during the initiation of atresia in pig follicles. *Domestic Anim. Endocrinol.* 72:106401 10.1016/j.domaniend.2019.106401 32278256

[B22] Hocking P. M. (2009). *Biology of Breeding Poultry.* Bodmin: CAB International.

[B23] Huttenhower C. Haley E. M. Hibbs M. A. Dumeaux V. Barrett D. R. Coller H. A. (2009). Exploring the human genome with functional maps. *Genome Res.* 19 1093–1106. 10.1101/gr.082214.108 19246570 PMC2694471

[B24] Jeck W. R. Sorrentino J. A. Wang K. Slevin M. K. Burd C. E. Liu J. (2013). Circular RNAs are abundant, conserved, and associated with ALU repeats. *RNA* 19 141–157. 10.1261/rna.035667.112 23249747 PMC3543092

[B25] Jia W. Xu B. Wu J. (2018). Circular RNA expression profiles of mouse ovaries during postnatal development and the function of circular RNA epidermal growth factor receptor in granulosa cells. *Metabolism* 85 192–204. 10.1016/j.metabol.2018.04.002 29634953

[B26] Jia Y. Lin J. Zeng W. Zhang C. (2010). Effect of prostaglandin on luteinizing hormone-stimulated proliferation of theca externa cells from chicken prehierarchical follicles. *Prostaglandins Other Lipid Mediat.* 92 77–84. 10.1016/j.prostaglandins.2010.03.005 20381633

[B27] Jia Y. Yang M. Zhu K. Wang L. Song Y. Wang J. (2016). Melatonin implantation improved the egg-laying rate and quality in hens past their peak egg-laying age. *Sci. Rep.* 6:39799 10.1038/srep39799 PMC5180240 28008984

[B28] Jing R. Gu L. Li J. Gong Y. (2018). A transcriptomic comparison of theca and granulosa cells in chicken and cattle follicles reveals ESR2 as a potential regulator of CYP19A1 expression in the theca cells of chicken follicles. *Comp. Biochem. Physiol. D Genomics Proteomics* 27 40–53. 10.1016/j.cbd.2018.04.002 29772405

[B29] Johnson A. L. (1990). Steroidogenesis and action of steroids in the hen ovary. *Crit. Rev. Poult. Biol.* 2 319–346.

[B30] Johnson A. L. (2003). Intracellular mechanisms regulating cell survival in ovarian follicles. *Anim. Reprod. Sci.* 78 185–201. 10.1016/s0378-4320(03)00090-3 12818644

[B31] Johnson A. L. (2015). Ovarian follicle selection and granulosa cell differentiation. *Poult. Sci.* 94 781–785. 10.3382/ps/peu008 25535403

[B32] Johnson A. L. Bridgham J. T. Wagner B. (1996). Characterization of a chicken luteinizing hormone receptor (cLH-R) complementary deoxyribonucleic acid, and expression of cLH-R messenger ribonucleic acid in the ovary. *Biol. Reprod.* 55 304–309. 10.1095/biolreprod55.2.304 8828833

[B33] Johnson A. L. Bridgham J. T. Woods D. C. (2004). Cellular mechanisms and modulation of activin A- and transforming growth factor beta-mediated differentiation in cultured hen granulosa cells. *Biol. Reprod.* 71 1844–1851. 10.1095/biolreprod.104.032573 15269104

[B34] Johnson P. A. (2012). Follicle selection in the avian ovary. *Reprod. Domest. Anim.* 47(Suppl. 4), 283–287. 10.1111/j.1439-0531.2012.02087.x 22827382

[B35] Kang L. Yang C. Wu H. Chen Q. Huang L. Li X. (2017). miR-26a-5p Regulates TNRC6A expression and facilitates theca cell proliferation in chicken ovarian follicles. *DNA Cell Biol.* 36 922–929. 10.1089/dna.2017.3863 28876086

[B36] Kim D. Johnson A. L. (2018). Differentiation of the granulosa layer from hen prehierarchal follicles associated with follicle-stimulating hormone receptor signaling. *Mol. Reprod. Dev.* 85 729–737. 10.1002/mrd.23042 29995345

[B37] Kim D. Lee J. Johnson A. L. (2016). Vascular endothelial growth factor and angiopoietins during hen ovarian follicle development. *Gen. Comp. Endocrinol.* 232 25–31. 10.1016/j.ygcen.2015.11.017 26996428

[B38] Kim D. Paggi J. M. Park C. Bennett C. Salzberg S. L. (2019). Graph-based genome alignment and genotyping with HISAT2 and HISAT-genotype. *Nat. Biotechnol.* 37 907–915. 10.1038/s41587-019-0201-4 31375807 PMC7605509

[B39] Langfelder P. Horvath S. (2008). WGCNA: an R package for weighted correlation network analysis. *BMC Bioinformatics* 9:559 10.1186/1471-2105-9-559 PMC2631488 19114008

[B40] Langmead B. Salzberg S. L. (2012). Fast gapped-read alignment with Bowtie 2. *Nat. Methods* 9 357–359. 10.1038/nmeth.1923 22388286 PMC3322381

[B41] Lebedev V. A. Lebedeva I. Y. Grossmann R. Kuzmina T. I. Parvizi N. (2006). Ovulatory cycle-related alterations in the thecal growth and membrane protein content of thecal tissue of hen preovulatory follicles. *Theriogenology* 66 217–223. 10.1016/j.theriogenology.2005.11.004 16325901

[B42] Lee K. A. Volentine K. K. Bahr J. M. (1998). Two steroidogenic pathways present in the chicken ovary: theca layer prefers delta 5 pathway and granulosa layer prefers delta 4 pathway. *Domest. Anim. Endocrinol.* 15 1–8. 10.1016/s0739-7240(97)00057-x 9437580

[B43] Li L. Guo J. Chen Y. Chang C. Xu C. (2017). Comprehensive CircRNA expression profile and selection of key CircRNAs during priming phase of rat liver regeneration. *BMC Genomics* 18:80 10.1186/s12864-016-3476-6 PMC5237265 28086788

[B44] Lin X. Liu X. Ma Y. Mi Y. Zeng W. Li J. (2018). Coherent apoptotic and autophagic activities involved in regression of chicken postovulatory follicles. *Aging* 10 819–832. 10.18632/aging.101436 29706614 PMC5940126

[B45] Liu G. Yang G. Chang B. Mercado-Uribe I. Huang M. Zheng J. (2010). Stanniocalcin 1 and ovarian tumorigenesis. *J. Natl. Cancer Inst.* 102 812–827. 10.1093/jnci/djq127 20484106 PMC2879417

[B46] Love M. I. Huber W. Anders S. (2014). Moderated estimation of fold change and dispersion for RNA-seq data with DESeq2. *Genome Biol.* 15;550. 10.1186/s13059-014-0550-8 PMC4302049 25516281

[B47] Lu T. Cui L. Zhou Y. Zhu C. Fan D. Gong H. (2015). Transcriptome-wide investigation of circular RNAs in rice. *RNA* 21 2076–2087. 10.1261/rna.052282.115 26464523 PMC4647462

[B48] Luo C.-W. Kawamura K. Klein C. Hsueh A. J. W. (2004). Paracrine regulation of ovarian granulosa cell differentiation by stanniocalcin (STC) 1: mediation through specific STC1 receptors. *Mol. Endocrinol.* 18 2085–2096. 10.1210/me.2004-0066 15131261

[B49] Lussier J. G. Diouf M. N. Lévesque V. Sirois J. Ndiaye K. (2017). Gene expression profiling of upregulated mRNAs in granulosa cells of bovine ovulatory follicles following stimulation with hCG. *Reprod. Biol. Endocrinol.* 15:88 10.1186/s12958-017-0306-x PMC5670713 29100496

[B50] McKeehan W. L. McKeehan K. A. (1979). Oxocarboxylic acids, pyridine nucleotide-linked oxidoreductases and serum factors in regulation of cell proliferation. *J. Cell Physiol.* 101 9–16. 10.1002/jcp.1041010103 575536

[B51] Mellouk N. Rame C. Barbe A. Grandhaye J. Froment P. Dupont J. (2018). Chicken is a useful model to investigate the role of adipokines in metabolic and reproductive diseases. *Int. J. Endocrinol.* 2018;4579734 10.1155/2018/4579734 PMC6029501 30018639

[B52] Memczak S. Jens M. Elefsinioti A. Torti F. Krueger J. Rybak A. (2013). Circular RNAs are a large class of animal RNAs with regulatory potency. *Nature* 495 333–338. 10.1038/nature11928 23446348

[B53] Nibbe R. K. Koyuturk M. Chance M. R. (2010). An integrative -omics approach to identify functional sub-networks in human colorectal cancer. *PLoS Comput. Biol.* 6:e1000639 10.1371/journal.pcbi.1000639 PMC2797084 20090827

[B54] Ouyang H. Chen X. Wang Z. Yu J. Jia X. Li Z. (2017). Circular RNAs are abundant and dynamically expressed during embryonic muscle development in chickens. *DNA Res.* 25 71–86. 10.1093/dnares/dsx039 PMC5824844 29036326

[B55] Paradis F. Novak S. Murdoch G. K. Dyck M. K. Dixon W. T. Foxcroft G. R. (2009). Temporal regulation of BMP2, BMP6, BMP15, GDF9, BMPR1A, BMPR1B, BMPR2 and TGFBR1 mRNA expression in the oocyte, granulosa and theca cells of developing preovulatory follicles in the pig. *Reproduction* 138 115–129. 10.1530/rep-08-0538 19359354

[B56] Peng Y. Chang L. Wang Y. Wang R. Hu L. Zhao Z. (2018). Genome-wide differential expression of long noncoding RNAs and mRNAs in ovarian follicles of two different chicken breeds. *Genomics* 111 1395–1403. 10.1016/j.ygeno.2018.09.012 30268779

[B57] Pertea M. Kim D. Pertea G. M. Leek J. T. Salzberg S. L. (2016). Transcript-level expression analysis of RNA-seq experiments with HISAT, StringTie and Ballgown. *Nat. Protoc.* 11 1650–1667. 10.1038/nprot.2016.095 27560171 PMC5032908

[B58] Picelli S. (2017). Single-cell RNA-sequencing: the future of genome biology is now. *RNA Biol.* 14 637–650. 10.1080/15476286.2016.1201618 27442339 PMC5449089

[B59] Qin Y. Iwase A. Murase T. Bayasula, Ishida C. Kato N. (2018). Protective effects of mangafodipir against chemotherapy-induced ovarian damage in mice. *Reprod. Biol. Endocrinol.* 16:106 10.1186/s12958-018-0426-y PMC6204278 30368246

[B60] Quan G. Li J. (2018). Circular RNAs: biogenesis, expression and their potential roles in reproduction. *J. Ovarian Res.* 11:9 10.1186/s13048-018-0381-4 PMC5773157 29343298

[B61] Rybak-Wolf A. Stottmeister C. Glazar P. Jens M. Pino N. Giusti S. (2015). Circular RNAs in the mammalian brain are highly abundant, conserved, and dynamically expressed. *Mol. Cell* 58 870–885. 10.1016/j.molcel.2015.03.027 25921068

[B62] Salzman J. Chen R. E. Olsen M. N. Wang P. L. Brown P. O. (2013). Cell-type specific features of circular RNA expression. *PLoS Genet.* 9:e1003777 10.1371/journal.pgen.1003777 PMC3764148 24039610

[B63] Salzman J. Gawad C. Wang P. L. Lacayo N. Brown P. O. (2012). Circular RNAs are the predominant transcript isoform from hundreds of human genes in diverse cell types. *PLoS One* 7:e30733 10.1371/journal.pone.0030733 PMC3270023 22319583

[B64] Sane A. Seidman E. Spahis S. Lamantia V. Garofalo C. Montoudis A. (2015). New insights in intestinal Sar1B GTPase regulation and role in cholesterol homeostasis. *J. Cell Biochem.* 116 2270–2282. 10.1002/jcb.25177 25826777

[B65] Seol H. S. Sato K. Murakami H. Toyomizu M. Akiba Y. (2006). Changes in gene expression involved in energy utilization during chicken follicle development. *Anim. Reprod. Sci.* 95 283–294. 10.1016/j.anireprosci.2005.09.016 16253445

[B66] Shang Q. Yang Z. Jia R. Ge S. (2019). The novel roles of circRNAs in human cancer. *Mol. Cancer* 18:6 10.1186/s12943-018-0934-6 PMC6325800 30626395

[B67] Shannon P. Markiel A. Ozier O. Baliga N. S. Wang J. T. Ramage D. (2003). Cytoscape: a software environment for integrated models of biomolecular interaction networks. *Genome Res.* 13 2498–2504. 10.1101/gr.1239303 14597658 PMC403769

[B68] Sirotkin A. V. (2011). Growth factors controlling ovarian functions. *J. Cell Physiol.* 226 2222–2225. 10.1002/jcp.22588 21660945

[B69] Stephens C. S. Johnson P. A. (2016). Bone morphogenetic protein 15 may promote follicle selection in the hen. *Gen. Comp. Endocrinol.* 235 170–176. 10.1016/j.ygcen.2016.06.027 27340039

[B70] Stephens C. S. Johnson P. A. (2017). Occludin expression and regulation in small follicles of the layer and broiler breeder hen. *Gen. Comp. Endocrinol.* 248 106–113. 10.1016/j.ygcen.2017.02.010 28238709

[B71] Storr S. J. Lee K. W. Woolston C. M. Safuan S. Green A. R. Macmillan R. D. (2012). Calpain system protein expression in basal-like and triple-negative invasive breast cancer. *Ann. Oncol. Off. J. Eur. Soc. Med. Oncol.* 23 2289–2296. 10.1093/annonc/mds176 PMC3425372 22745213

[B72] Sutherland J. M. Keightley R. A. Nixon B. Roman S. D. Robker R. L. Russell D. L. (2012). Suppressor of cytokine signaling 4 (SOCS4): moderator of ovarian primordial follicle activation. *J. Cell. Physiol.* 227 1188–1198. 10.1002/jcp.22837 21604262

[B73] Tao H. Xiong Q. Zhang F. Zhang N. Liu Y. Suo X. (2017). Circular RNA profiling reveals chi_circ_0008219 function as microRNA sponges in pre-ovulatory ovarian follicles of goats (Capra hircus). *Genomics* [Epub ahead of print] 10.1016/j.ygeno.2017.10.005 29107014

[B74] Tay Y. Rinn J. Pandolfi P. P. (2014). The multilayered complexity of ceRNA crosstalk and competition. *Nature* 505 344–352. 10.1038/nature12986 24429633 PMC4113481

[B75] Tilly J. L. Kowalski K. I. Johnson A. L. (1991a). Stage of ovarian follicular development associated with the initiation of steroidogenic competence in avian granulosa cells. *Biol. Reprod.* 44 305–314. 10.1095/biolreprod44.2.305 1849025

[B76] Tilly J. L. Kowalski K. I. Johnson A. L. Hsueh A. J. (1991b). Involvement of apoptosis in ovarian follicular atresia and postovulatory regression. *Endocrinology* 129 2799–2801. 10.1210/endo-129-5-2799 1718732

[B77] Toyota M. Suzuki H. Sasaki Y. Maruyama R. Imai K. Shinomura Y. (2008). Epigenetic silencing of microRNA-34b/c and B-cell translocation gene 4 is associated with CpG island methylation in colorectal cancer. *Cancer Res.* 68 4123–4132. 10.1158/0008-5472.can-08-0325 18519671

[B78] Trapnell C. Williams B. A. Pertea G. Mortazavi A. Kwan G. van Baren M. J. (2010). Transcript assembly and quantification by RNA-Seq reveals unannotated transcripts and isoform switching during cell differentiation. *Nat. Biotechnol.* 28 511–515. 10.1038/nbt.1621 20436464 PMC3146043

[B79] Uri-Belapolsky S. Miller I. Shaish A. Levi M. Harats D. Ninio-Many L. (2017). Interleukin 1-alpha deficiency increases the expression of Follicle-stimulating hormone receptors in granulosa cells. *Mol. Reprod. Dev.* 84 460–467. 10.1002/mrd.22799 28337831

[B80] Wang J. Gong Y. (2017). Transcription of CYP19A1 is directly regulated by SF-1 in the theca cells of ovary follicles in chicken. *Gen. Comp. Endocrinol.* 247 1–7. 10.1016/j.ygcen.2017.03.013 28347743

[B81] Woods D. C. Schorey J. S. Johnson A. L. (2009). Toll-like receptor signaling in hen ovarian granulosa cells is dependent on stage of follicle maturation. *Reproduction* 137 987–996. 10.1530/rep-08-0320 19336472

[B82] Wulff C. Wiegand S. J. Saunders P. T. Scobie G. A. Fraser H. M. (2001). Angiogenesis during follicular development in the primate and its inhibition by treatment with truncated Flt-1-Fc (vascular endothelial growth factor Trap(A40)). *Endocrinology* 142 3244–3254. 10.1210/endo.142.7.8258 11416048

[B83] Xie C. Mao X. Huang J. Ding Y. Wu J. Dong S. (2011). KOBAS 2.0: a web server for annotation and identification of enriched pathways and diseases. *Nucleic Acids Res.* 39 W316–W322. 10.1093/nar/gkr483 21715386 PMC3125809

[B84] Xu T. Wu J. Han P. Zhao Z. Song X. (2017). Circular RNA expression profiles and features in human tissues: a study using RNA-seq data. *BMC Genomics* 18(Suppl. 6):680. 10.1186/s12864-017-4029-3 PMC5629547 28984197

[B85] Yamamoto N. Christenson L. K. McAllister J. M. Strauss J. F. III (2002). Growth differentiation factor-9 inhibits 3’5’-adenosine monophosphate-stimulated steroidogenesis in human granulosa and theca cells. *J. Clin. Endocrinol. Metab.* 87 2849–2856. 10.1210/jcem.87.6.8551 12050262

[B86] Yao H. H. Bahr J. M. (2001). Chicken granulosa cells show differential expression of epidermal growth factor (EGF) and luteinizing hormone (LH) receptor messenger RNA and differential responsiveness to EGF and LH dependent upon location of granulosa cells to the germinal disc. *Biol. Reprod.* 64 1790–1796. 10.1095/biolreprod64.6.1790 11369610

[B87] Yao X. Zhang G. Guo Y. Ei-Samahy M. Wang S. Wan Y. (2017). Vitamin D receptor expression and potential role of vitamin D on cell proliferation and steroidogenesis in goat ovarian granulosa cells. *Theriogenology* 102 162–173. 10.1016/j.theriogenology.2017.08.002 28797922

[B88] Yoshimura Y. Koga O. (1982). Ultrastructural changes of the stigma of the follicle during the process of ovulation in the hen. *Cell Tissue Res.* 224 349–359. 10.1007/bf00216878 7105137

[B89] You X. Vlatkovic I. Babic A. Will T. Epstein I. Tushev G. (2015). Neural circular RNAs are derived from synaptic genes and regulated by development and plasticity. *Nat. Neurosci.* 18 603–610. 10.1038/nn.3975 25714049 PMC4376664

[B90] Yu J. Lou Y. Zhao A. (2016). Transcriptome analysis of follicles reveals the importance of autophagy and hormones in regulating broodiness of Zhedong white goose. *Sci. Rep.* 6:36877 10.1038/srep36877 PMC5105085 27833138

[B91] Zhang X. Yan Y. Lei X. Li A. Zhang H. Dai Z. (2017). Circular RNA alterations are involved in resistance to avian leukosis virus subgroup-J-induced tumor formation in chickens. *Oncotarget* 8 34961–34970. 10.18632/oncotarget.16442 28415618 PMC5471026

[B92] Zhang Y. L. Xia Y. Yu C. Richards J. S. Liu J. Fan H. Y. (2014). CBP-CITED4 is required for luteinizing hormone-triggered target gene expression during ovulation. *Mol. Hum. Reprod.* 20 850–860. 10.1093/molehr/gau040 24878634 PMC4131766

[B93] Zhou L. Chen J. Li Z. Li X. Hu X. Huang Y. (2010). Integrated profiling of microRNAs and mRNAs: microRNAs located on Xq27.3 associate with clear cell renal cell carcinoma. *PLoS One* 5:e15224 10.1371/journal.pone.0015224 PMC3013074 21253009

[B94] Zhu G. Kang L. Wei Q. Cui X. Wang S. Chen Y. (2014). Expression and regulation of MMP1, MMP3, and MMP9 in the chicken ovary in response to gonadotropins, sex hormones, and TGFB1. *Biol. Reprod.* 90:57 10.1095/biolreprod.113.114249 24451989

[B95] Zhu G. Mao Y. Zhou W. Jiang Y. (2015). Dynamic changes in the follicular transcriptome and promoter DNA methylation pattern of steroidogenic genes in chicken follicles throughout the ovulation cycle. *PLoS One* 10:e0146028 10.1371/journal.pone.0146028 PMC4696729 26716441

