# Supplementary figures and images for: Transcriptomic Analysis of circRNAs and mRNAs Reveals a Complex Regulatory Network That Participate in Follicular Development in Chickens
Source: Front Genet. 2020 May 15;11:503. doi: 10.3389/fgene.2020.00503 (PMC7243251; doi:10.3389/fgene.2020.00503)

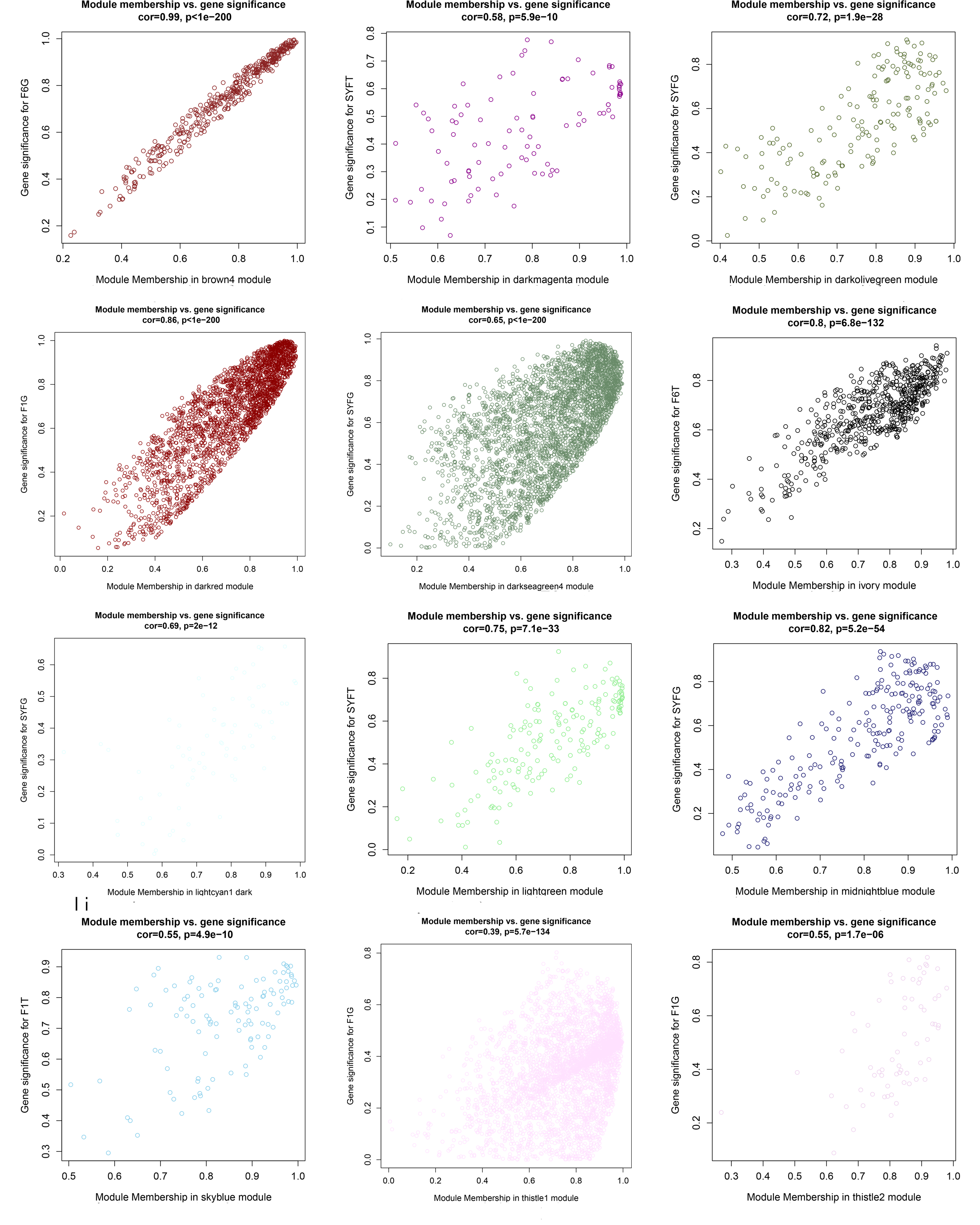

Supplement: FIGURE S1 — Visualization of GS vs. MM. The scatter plots display the distribution of GS and MM of transcripts in stage-specific modules related to individual sample. [file Image_1.TIF]
